# Seasonal variation in wind speed and oceanic salt spray favors delayed reproduction in coastal yellow monkeyflowers

**DOI:** 10.1002/ajb2.70249

**Published:** 2026-08-04

**Authors:** Katherine Toll, Chloe Crockett, Lauren Tocci, David B. Lowry

**Affiliations:** ^1^ Department of Biological Sciences University of South Carolina Columbia 29208 SC USA; ^2^ Department of Plant Biology Michigan State University East Lansing 48824 MI USA; ^3^ Plant Resilience Institute, Michigan State University East Lansing 48824 MI USA

**Keywords:** adaptation, Bodega Marine Reserve, coastal, flowering time, monkeyflower, Phrymaceae, salt spray, wind

## Abstract

**Premise:**

The optimal timing of reproduction depends on the relative risk and benefit of continued growth. These risks and benefits depend on the local environment, which varies seasonally. While temperature and water availability are well‐studied selective agents on reproductive timing, less is known about other seasonally variable factors.

**Methods:**

To test whether seasonal variation in wind and salt spray influences selection on reproductive timing, we manipulated flowering time and ocean exposure in a coastal environment. Flowering plants from 12 yellow monkeyflower populations that span a coastal‐to‐inland gradient were placed on a coastal bluff in spring and summer. Inland populations typically flower in spring, which is much windier than the summer months when coastal populations usually flower, other plants were protected from wind and salt spray, and the rest were exposed to wind and salt spray.

**Results:**

In the windier spring cohort, individuals exposed to the ocean had severe necrosis and produced few flowers and seeds, while protected plants had little to no necrosis, producing more flowers and seeds than ocean‐exposed plants. In the calmer summer cohort, individuals exposed to the ocean had less severe necrosis and produced comparable numbers of flowers as plants protected from the ocean. However, only coastal populations produced appreciable numbers of seeds when exposed to the ocean, consistent with prior evidence that coastal ecotypes are locally adapted.

**Conclusions:**

Overall, these results suggest that seasonal variation in wind and salt spray is a key selective factor in coastal populations contributing to population differences in flowering time.

The timing of reproduction is a key life‐history transition that affects fitness. Life history theory predicts that the optimal timing of reproduction should depend on the costs of continued growth versus the benefits of growing for longer (Cohen, 1976; Kozłowski, 1992). Costs of continued growth include an increased risk of tissue loss or death before reproduction, while the benefits include an increase in size and offspring number. These relative risks and benefits depend on an organism's environment, which is rarely constant. Seasonal variation in temperature, daylength, and water and resource availability can limit the growing season and are major drivers of variation in the timing of life‐history transitions across environments and over time (Rathcke and Lacey, 1985; Cleland et al., 2007). While temperature and water availability are the best studied, other local factors also determine an organism's growing season and could generate divergent selection on reproductive timing across environments.

Organisms inhabiting coastal environments experience several unique abiotic stressors relative to inland environments, including exposure to wave action (Connell, 1972), oceanic salt spray, sand abrasion, and sand burial (DiVittorio et al., 2020). These stressors limit growth and often result in pronounced patterns of coastal vegetation zonation (Oosting and Billings, 1942). Salt spray is a dominant factor governing the distribution and abundance of plant species in coastal environments (Barbour and DeJong, 1977; Du and Hesp, 2020), although its direct role in the evolution of plant physiology, morphology, and phenology is less well understood (Ahmad and Wainwright, 1976; Itoh, 2021). Many plant lineages have evolved putative adaptations to coastal stressors, such as thick, succulent leaves and prostrate growth (Turesson, 1922; Boyce, 1954; Lowry, 2012), although similar phenotypes may be induced by salt spray (Wells and Shunk, 1938; Boyce, 1951; Griffiths, 2006). Since new reproductive structures are produced at apical meristems, above vegetative tissues where wind and salt spray exposure is higher, flowers and fruits are often more susceptible to damage than leaves. Damage to reproductive meristems is costly when meristems are limited (Geber, 1990), and this type of damage might favor the evolution of strategies to escape or tolerate recurrent injury (Wise and Abrahamson, 2008).

Populations of yellow monkeyflower [*Erythranthe guttata* (DC.) G.L. Nesom, (syn. *Mimulus guttatus* DC.) species complex; Phrymaceae] grow across a coastal‐to‐inland gradient and differ in life history, reproductive timing, and growth habit (Hall and Willis, 2006; Hall et al., 2006; Lowry and Willis, 2010). Monkeyflower populations in California and Oregon experience mild wet winters, and hot dry summers. Coastal populations are typically perennials with prostrate growth habits and tend to germinate in winter (but can germinate year round) and flower in summer, where fog is a source of soil moisture despite the lack of rainfall (Hall and Willis, 2006; Lowry et al., 2008). In contrast, inland populations growing near ephemeral water sources are typically annuals with erect growth habits that tend to germinate in winter and flower in spring, before the summer drought. Early flowering is favored in inland environments due to strong viability selection at the end of the growing season imposed by drought, while early flowering is disfavored in coastal environments due to resource allocation trade‐offs (Hall and Willis, 2006; Lowry et al., 2008). Since early flowering is often positively correlated with plant size, early‐flowering annuals do not build up sufficient vegetative growth necessary for continuous flowering throughout a longer growing season relative to later‐flowering perennials (Hall and Willis, 2006; Bolmgren and D. Cowan, 2008). However, resource allocation trade‐offs do not fully explain fitness differences between annuals and perennials at coastal sites. In reciprocal transplant experiments initiated in winter, annual transplants in coastal environments typically die before reproducing, but can survive to reproduce when transplanted in late spring (Hall and Willis, 2006; Lowry et al., 2008; Lowry and Willis, 2010; Popovic and Lowry, 2020; Toll et al., 2026). Therefore, we hypothesize that early‐season factors influence selection on flowering time in coastal environments. In our study region, coastal Northern California, wind speeds are generally higher during the winter–spring rainy season than in the relatively calmer summer and fall and are much higher near the coast (Gilliam, 2002; García‐Reyes and Largier, 2012). Coastal populations are exposed to oceanic salt spray produced by wave action and distributed by wind (García‐Reyes and Largier, 2012; Du and Hesp, 2020).

To test whether seasonal variation in wind and salt spray might be a selective agent contributing to heritable differences in life‐history timing between coastal and inland populations, we experimentally manipulated flowering time and ocean exposure. Since coastal perennial populations require substantially longer days to initiate flowering than annuals (Friedman and Willis, 2013), we induced flowering using supplemental light. We then placed plants (just before or after anthesis) outdoors in two temporal cohorts, aligned with when inland annual (spring) and coastal perennial (summer) plants flower at the same latitude. We protected a subset of plants from salt spray and wind in both seasons with exclosures and exposed the remaining plants in control structures. If coastal populations are able to escape tissue death and increase fitness by temporally escaping wind and salt spray, we predicted that (1) tissue death would be higher and fecundity would be lower during the windier spring cohort compared to the calmer summer cohort, (2) protection from wind and salt spray would reduce tissue death and increase fitness, and (3) the benefit of protection would be greater in the spring than in the summer.

## MATERIALS AND METHODS

We performed our experiments at the Bodega Marine Reserve in Bodega Bay, Sonoma County, California, United States (38.31841, –123.07329). To prevent gene flow between experimental and local plants, we placed our arrays near, but not in, natural seeps containing monkeyflowers. Our site was 0.24 km and 0.47 km from two natural seep populations and within a comparable distance to the ocean as these natural populations (under 50 m).

**Figure 1:**
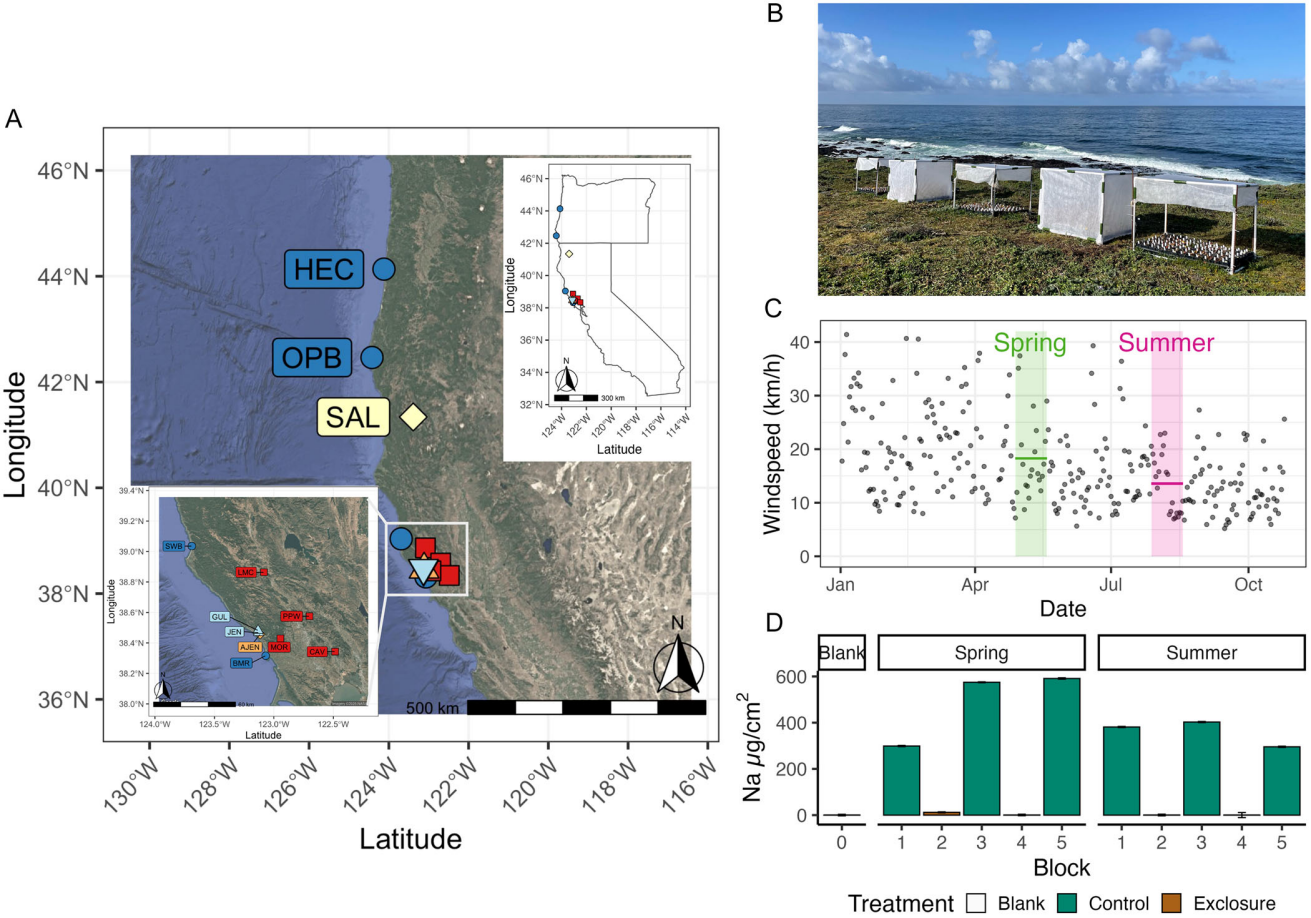
(A) Map of collection localities of ocean‐exposed coastal perennial (dark blue circles), inland annual (red squares), inland perennial (yellow diamond), ocean‐protected coastal annual (orange triangle), and ocean‐protected coastal perennial (light blue triangles) populations used in the experiment, (B) photograph of experiment at Bodega Marine Reserve (photo credit: K Toll), (C) plot of average daily wind speed at the Bodega Marine Reserve in 2023, and (D) plot of sodium accumulation on filter paper traps during the spring and summer exposure periods in exclosures (brown) and controls (green). Wind speeds were higher for the spring cohort than the summer cohort (C, pink vs. green), and exclosures significantly reduced Na accumulation in both seasons (D, green vs. brown).

We grew a single outbred full‐sib family from each of 12 localities spanning a coastal‐to‐inland gradient (Figure 1A; Appendix S1: Figure S1, Table S1). Outbred families were generated by intercrossing two randomly sampled individuals from each locality. We chose this breeding design because we were primarily interested in differences among accessions and because of strong inbreeding depression in this mixed‐mating species (Willis, 1993). Four accessions were inland annual ecotypes from Sonoma County, California, and six accessions were coastal perennial ecotypes spanning a latitudinal cline in plant height from Oregon to Sonoma County (Zambiasi and Lowry, 2024). All inland annual populations experience a terminal summer drought, and all coastal populations grow in persistent seeps and experience a maritime climate with coastal fog. Four of the coastal perennial ecotype accessions were collected from sites directly exposed to oceanic salt spray (exposed coastal perennial populations, following Zambiasi and Lowry, 2024), while two of the coastal perennial accessions were collected from sites not directly exposed to the ocean (protected coastal perennial populations, following Zambiasi and Lowry, 2024). In the field and in greenhouse common gardens, protected coastal perennial populations are taller on average than exposed coastal perennial populations, suggesting that prostrate growth may contribute to the avoidance of salt spray damage (Zambiasi and Lowry, 2024). We also grew an inland perennial accession from a large river in Siskiyou County, California. Lastly, we included an ocean‐protected coastal annual accession that was collected less than 1.6 km from the protected coastal perennial accessions. The protected coastal annual accession was collected from a rocky serpentine seep in a coastal headland and thus experiences a maritime climate without exposure to salt spray, but with terminal drought due to the low water‐holding capacity of serpentine soils.

Since annuals germinate faster than perennials, we planted perennial and annual seeds on Sungro Sunshine Mix #4 (Agawam, MA, USA) a week apart in two seasons (spring and summer) (following the method of Popovic and Lowry, 2020) in the Bodega Marine Reserve greenhouse (spring perennials: 2 March 2023, annuals: 9 March 2023; summer perennials: 5 June 2023, annuals 12 June 2023). We evenly distributed nine seedlings per accession across five blocks of six flats, for a total of 45 seedlings per accession per cohort (540 plants per cohort). In each randomized block, six flats contained at least one seedling per accession, while two flats were randomly assigned a second seedling. Since spring daylengths at our study site were not long enough to induce flowering (Friedman and Willis, 2013), we grew plants under supplemental lights for 16 h per day in both seasons.

Once plants initiated flowering, roughly 2 months after seed planting, we moved flats from the greenhouse into five outdoor blocks on a coastal bluff. To protect plants from wind and salt spray outdoors in each cohort, we completely covered two of five blocks with agrofabric (Figure 1B). Agrofabric exclosures were 108 × 84 × 87 cm and constructed of PVC pipe (3/4 inch [19.05 mm]) covered with medium‐weight agrofabric (Pro‐34 1.0 oz/yd^2^ with 70% light transmission; OBC Northwest, Canby, OR, USA) affixed with hot glue and PVC clips. Each structure was secured to the ground with rebar attached to the PVC with zip‐ties. Exclosures had one door facing away from the ocean and secured with PVC clips to allow access to the plots. To control for agrofabric shading or moisture collection, we constructed the remaining three blocks identically, except that agrofabric only covered their tops and 30 cm down each side. In both seasons, we covered the control structures with bird netting to reduce herbivory. We did not observe herbivory in spring, but in summer, the inflorescences of a few plants on the edges of the control structures were eaten by mule deer (*Odocoileus hemionus*).

At the start of the outdoor period of each cohort, we measured plant height and removed petals from any open flowers. Plants were left outdoors for 3 weeks in each cohort (spring: 28 April to 19 May 2023, summer: 28 July to 18 August 2023). We censused plants daily and manually self‐fertilized up to three new flowers per plant during the outdoor period of each cohort. Thereafter, we removed petals from each new flower that opened daily outdoors to prevent gene flow with local populations. We recorded leaf and inflorescence necrosis (tissue death) on an ordinal scale (no, partial, or complete necrosis) weekly during the outdoor period of each cohort. Since we observed apical necrosis on plants exposed to the ocean in control structures, we measured living tissue height on each plant weekly. We bottom‐watered flats as needed. At the end of the 3‐week outdoor period of each cohort, we transported flats back into the greenhouse, where we collected the fruit we previously hand‐pollinated outdoors as they ripened (spring: 19 May to 13 June 2023, summer: 19 August to 12 September 2023). Seeds from each fruit were counted by eye.

To quantify wind speed in each season, we downloaded average daily wind speed data from the Bodega Ocean Observing Node, measured within meters of our experiment (https://boon.ucdavis.edu/data-access/products/met/bml_wind_speed_daily; University of California, Davis, Bodega Marine Laboratory, Bodega Bay, CA, USA). To quantify salt spray, we constructed filter paper traps with a 3D printer. The traps were 60 × 60 × 103 mm and had a 20 × 30 mm opening for filter paper on all four sides. We cut Whatman filter paper (qualitative, grade 2) in 38 × 60 mm pieces and placed them on three sides of each trap. We affixed the traps to the southeast corner of each structure, 380 mm from the ground, and oriented them so one side faced west at the Pacific Ocean. West‐facing filter paper samples were cut into a 1‐cm^2^ square using a pair of clean scissors that were washed between samples. The filter paper samples were then soaked in 10 mL of ultrapure 18 MOhm water for 72 h. A sample of that solution was taken out and spiked with indium (internal standard to correct for drift). The samples were analyzed by the University of South Carolina Center for Elemental Mass Spectrometry using a Thermo Scientific (Waltham, MA, USA) ELEMENT 2 high‐performance high‐resolution inductively coupled plasma mass spectrometer (HR‐ICPMS) in high‐resolution mode. Sodium concentrations were calculated with external calibration of Na pure solution, spiked with In.

### Statistical analyses

All statistical analyses were performed in R version 4.4.1 (R Core Team, 2024). Mixed models were analyzed using the R package glmmTMB (Brooks et al., 2017) or ordinal (Christensen, 2023), model term significance was assessed using analysis of deviance (Wald *χ* ^2^ test) with the R package car (Fox and Weisberg, 2018), and pairwise comparisons were conducted using the R package emmeans (Lenth et al., 2020). We predicted means and 95% confidence intervals using the R package ggeffects (Lüdecke, 2018).

To test whether necrosis severity differed between the agrofabric exclosure treatment and control and between seasons, we analyzed two necrosis proxies. Our first necrosis proxy was living tissue height over time, since we observed that plants in control structures in the spring cohort initially exhibited necrosis at their apical meristems and died back toward their bases. We first visualized height over time by plotting raw height values during each census. To evaluate initial differences in plant height across accessions and seasons, we analyzed height measured in the greenhouse (just before moving plants outdoors) using a linear mixed model with accession (*N* = 12), season (*N* = 2), and the two way interaction as fixed effects, and flat (*N* = 60) as a random effect.

To reduce model complexity (i.e., to avoid fitting a model with a four‐way accession, season, exclosure treatment, and days outdoors interaction), we calculated the change in living tissue height for each plant by subtracting the initial height measured in the greenhouse from the final height after 21 days outdoors. We analyzed the change in height using a linear mixed model with accession (*N* = 12), season (*N* = 2), and exclosure treatment (*N* = 2), all two‐way interactions, and the three‐way interaction as fixed effects, and flat (*N* = 60) nested within block (*N* = 10) as random effects. To understand how sensitive our analysis of the change in plant height was to initial plant height, we conducted two additional analyses. First, we analyzed the change in height over each seasonal cohort using the same model just described with two additional covariates: initial plant height and a height by season interaction. Next, we analyzed the change in height using the same terms as the first model but filtered our data set to only include individuals within each family that overlapped in initial height across seasonal cohorts (taller than the shortest plants in spring and shorter than the tallest plants in summer).

Our second necrosis proxy was a qualitative assessment of necrosis on leaves and inflorescences in three categories of severity (no, partial, or complete necrosis). We plotted the categories of severity over time and tested whether leaf or inflorescence necrosis at the end of each exposure period differed between seasons and treatments using a cumulative link mixed model, a method for analyzing ordinal response variables with fixed and random effects (Christensen, 2023). The necrosis severity model consisted of season and exclosure treatment as fixed effects, and block as a random effect since interactive models and models with flat nested within block were singular. We did not include accession in the necrosis severity model because there was no variance in severity for most accessions within each treatment and season, and thus the model was not able to estimate severity for those accession, season, and treatment combinations.

To test whether fecundity differed between the agrofabric exclosure treatment and control and between seasons, we analyzed flower production and seeds produced by up to three hand‐pollinated flowers per plant. We analyzed flowers per plant using a mixed model with a negative binomial error distribution since the Poisson model was overdispersed. The model consisted of season, accession, exclosure treatment, all two‐way interactions, and the three‐way interaction as fixed effects, and flat nested within block as random effects. To analyze seed production, we first averaged seeds produced by each plant to avoid pseudoreplication. We analyzed seeds per flower using a mixed model with a zero‐inflated negative binomial error distribution since the Poisson model was significantly overdispersed. Because the model with a three‐way interaction did not converge, the seed model consisted of season, accession, exclosure treatment, and all two‐way interactions as fixed effects, and flat nested within block as random effects.

## RESULTS

Average daily wind speeds were 5 km/h faster during the spring cohort compared to the summer cohort (spring mean, range: 18.3, 7.2–37.4 km/h; summer mean, range: 13.6, 7.0–23.0 km/h; Figure 1C). Sodium (Na) deposition in the three control structures ranged from 298.9–591.3 µg/cm^2^ in spring and 295.6–402.8 µg/cm^2^ in summer (Figure 1D). In each season, the two exclosures had little to no Na deposition (12.0 and 0.6 µg/cm^2^ in spring; 0.2 and 0.4 µg/cm^2^ in summer), comparable to a blank filter paper control (0.2 µg/cm^2^). Agrofabric exclosures effectively reduced Na accumulation (Figure 1D, 242 vs. 3.3 µg/cm^2^, two‐tailed *t*‐test: *t* = 7.89, df = 5.03, *P* < 0.001). An intense spring storm briefly detached the bottom northwest corner of agrofabric covering one of the exclosures, reflected by a slightly higher Na concentration (12.0 µg/cm^2^) in that block, which is still 25 times lower than in the control blocks. We promptly secured the agrofabric with additional adhesive and PVC clips after the storm.

Initial height in the greenhouse was significantly associated with accession (*χ* ^2^ = 1482.13, df = 11, *P* < 0.001), season (*χ* ^2^ = 129.55, df = 1, *P* < 0.001), and an accession by season interaction (*χ* ^2^ = 903.57, df = 11, *P* < 0.001). All inland annuals and ocean‐exposed coastal perennials differed in initial height in the spring cohort, but not in the summer cohort (Tukey post hoc contrasts; Appendix S1: Table S2). Just before the plants were placed outdoors in spring, the tallest families were three of the four inland annuals (all > 210 mm), followed by the inland perennial, one of the ocean‐protected coastal perennials, the ocean‐protected coastal annual and one of the inland annuals (all > 161 mm); the remaining protected (*N* = 1) and exposed (*N* = 4) coastal perennial ecotypes were shorter than 97 mm. Just before plants were placed outdoors in summer, the tallest plants initially were the inland perennial (222 mm), followed by one of the inland annuals (180 mm) and one of the exposed coastal perennials (131 mm); all other accessions were initially shorter than 66 mm. Seven of the 12 accessions (four inland annuals, the ocean‐protected coastal annual, and two ocean‐protected coastal perennials) were 28–83% taller initially in spring compared to summer (Appendix S1: Table S2, Tukey post hoc comparisons of spring versus summer height within accessions, all *P* < 0.001). Two of the 12 accessions (an inland perennial and exposed coastal perennial) were 22–35% shorter initially in spring compared to summer (Appendix S1: Table S2, Tukey post hoc comparisons of spring versus summer height within each accession, both *P* < 0.025). Finally, three of the 12 accessions (three ocean‐exposed coastal perennials) did not significantly differ in initial height across seasons (Appendix S1: Table S2, Tukey post hoc comparisons all *P* > 0.96).

**Figure 2:**
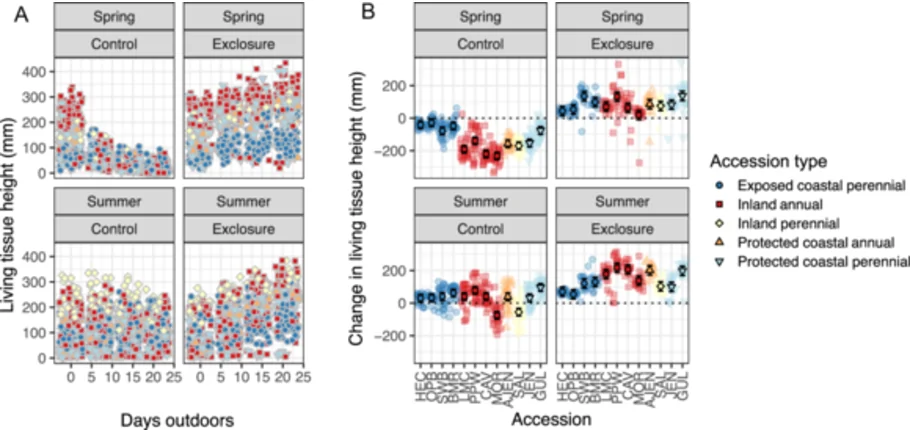
Spring‐flowering plants had severe apical tissue death when exposed to the ocean, reflected by the decrease in living tissue height, and agrofabric exclosures protected plants from necrosis. (A) Living tissue height over time, measured before moving plants outside and at three weekly censuses in ocean‐exposed controls or agrofabric protected exclosures. (B) Individual height differences (final minus initial height) (background points) and predictions from a linear mixed model (black outlined points; mean and 95% confidence intervals).

Necrosis, estimated by change in living tissue height, was significantly associated with accession, season, exclosure treatment, an accession by season interaction, an accession by treatment interaction, and the three‐way accession by season by exclosure treatment interaction (all *P* < 0.001, Table 1). In spring, when wind speeds were highest (Figure 1C), plants exposed to the ocean in control structures had severe apical tissue death, reflected by a significant decrease in living tissue height in the control structures (Figure 2). In summer, we did not observe extreme apical necrosis, and most plants in the control structures increased in height over time (Figure 2), but not as much as in the agrofabric exclosures (all *P* ≤ 0.04, Appendix S1: Table S3). In both seasons, plants in the agrofabric exclosures increased in height (Figure 2), and the change in height significantly differed between agrofabric exclosures and controls (all *P* < 0.001, Appendix S1: Table S3). In the control structures, three of the four ocean‐exposed coastal perennials, two of the four inland annuals, both ocean‐protected coastal perennials, and the ocean‐protected coastal annual had greater tissue necrosis (more negative growth) in spring compared to summer (all *P* < 0.05, Appendix S1: Table S2). Including initial height and a season by initial height interaction as a covariate or subsetting the data to only include individuals within each accession that overlapped in initial height, did not change the overall results: Effects were significant and in the same direction as the initial analysis even if the magnitude differed. For example, when we included the initial height covariates, inland annuals decreased in living tissue height in spring and increased in living tissue height in summer, but the predicted change was smaller than in the model without covariates. In that same model, inland annuals increased in living tissue height in the spring agrofabric exclosures, but the predicted change was greater than in the model without covariates. When we subset the data, the change in inland annual height was more variable, but generally decreased in the control structures and increased in the exclosures. Detailed results can be found in Appendix S2.

**Table 1:** Analysis of deviance tables (Type III Wald *χ* ^2^ tests) for change in living tissue height, flower production, and seed production.

Response	Factor	χ^2^	df	*P*
Change in height	(Intercept)	11.9221	1	0.0005547
	Accession	545.0121	11	<2.2E‐16
	Season	17.7024	1	2.58E‐05
	Treatment	20.3557	1	6.43E‐06
	Accession:Season	219.1006	11	<2.2E‐16
	Accession:Treatment	206.2958	11	<2.2E‐16
	Season:Treatment	2.8391	1	0.0919932
	Accession:Season:Treatment	33.961	11	0.000367
Leaf necrosis	Season	73.622	1	<2.2E‐16
	Treatment	129.923	1	<2.2E‐16
Inflorescence necrosis	Season	204.87	1	<2.2E‐16
	Treatment	362.99	1	<2.2E‐16
No. flowers/plant	(Intercept)	11.788	1	0.0005962
	Season	44.699	1	2.30E‐11
	Accession	92.857	11	4.58E‐15
	Treatment	54.457	1	1.59E‐13
	Season:Accession	30.849	11	0.0011638
	Season:Treatment	25.86	1	3.67E‐07
	Accession:Treatment	104.645	11	<2.2E‐16
	Season:Accession:Treatment	35.268	11	0.0002238
No. seeds/flower	(Intercept)	149.612	1	<2.2E‐16
	Season	120.804	1	<2.2E‐16
	Treatment	154.401	1	<2.2E‐16
	Accession	361.07	11	<2.2E‐16
	Season:Treatment	133.033	1	<2.2E‐16
	Season:Accession	25.824	11	0.006892
	Treatment:Accession	300.598	11	<2.2E‐16

Leaf and inflorescence necrosis were both significantly associated with season (*P* < 0.001) and exclosure treatment (*P* < 0.001, Table 1). By the end of the spring cohort, 97–99% of plants exposed to the ocean in control structures had complete leaf (314/324) and inflorescence (323/324) necrosis (i.e., no living tissue remained), while the remainder had partial necrosis (some living tissue) (Appendix S1: Figures S2, S3a, S4; Table S4). By the end of the summer cohort, 78–83% of plants in the control structures had partial leaf (268/324) and inflorescence (253/324) necrosis, while the remainder had no necrosis (Appendix S1: Figure S3b). A small percentage (9–12%) of plants, near the northwest corners of the spring agrofabric exclosures had leaf (26/216) and inflorescence (20/216) necrosis due to the intense storm that briefly detached the protective agrofabric.

**Figure 3:**
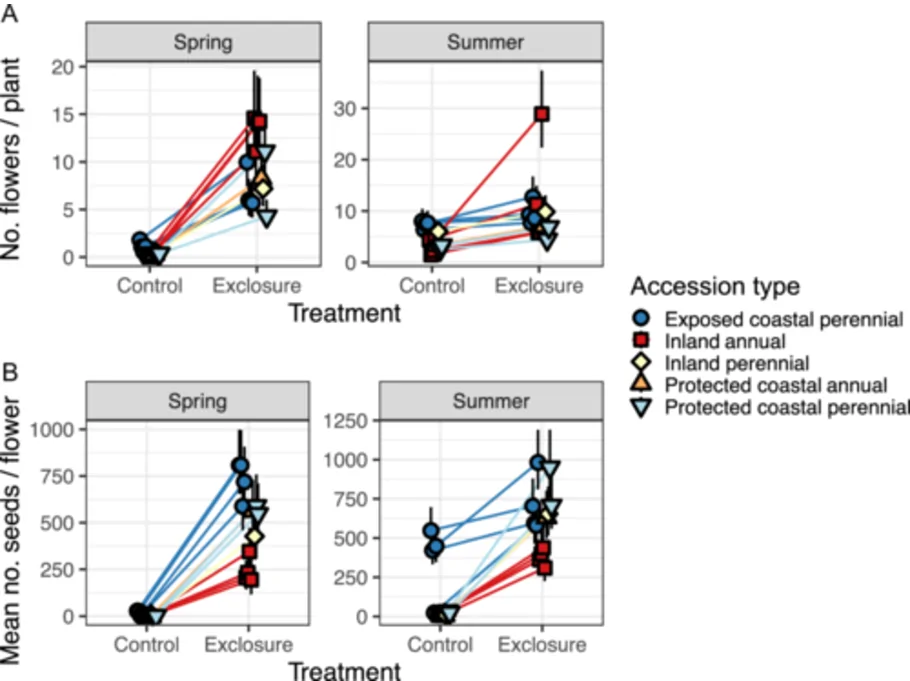
Plants produced fewer flowers per plant (A) and seeds per flower (B) in spring than in summer when exposed to the ocean, plants protected by agrofabric exclosures produced more flowers and seeds than those exposed to the ocean, and the benefit protection was greater in spring. (A) Number of predicted flowers per plant (mean and 95% confidence intervals) and (B) number of predicted seeds per flower (mean and 95% confidence intervals) from mixed models.

Flower production was significantly associated with season, accession, exclosure treatment, and all two‐way and the three‐way interactions (all *P* < 0.001; Table 1; Figure 3A). In spring, all accessions exposed to the ocean in control structures produced significantly fewer flowers than those protected in agrofabric exclosures (4–14 fewer flowers, all *P* < 0.001; Appendix S1: Table S5, Figure S5A, S6). In summer, some (7/12) accessions in the control structures produced significantly fewer flowers than those in agrofabric exclosures, and the difference was smaller (2–7 fewer flowers except one inland annual that made 24 fewer flowers, *P* ≤ 0.05; Appendix S1: Table S5, Figure S5A). Accessions exposed to the ocean in control structures produced 2–7 fewer flowers in spring compared to summer (*P* < 0.001, Appendix S1: Table S4, Figure S5A). Most (8/12) accessions in agrofabric exclosures did not differ in flower number between seasons, and significant differences varied in direction (–14 to +8 in spring vs summer, Appendix S1: Table S5, Figure S5A).

Average seed production per hand‐pollinated flower was significantly associated with season, accession, exclosure treatment, and all two‐way interactions (all *P* ≤ 0.006; Table 1, Figure 3B). In spring, only coastal perennials from ocean‐exposed localities produced seeds when exposed to the ocean in the control structures (Appendix S1: Figure S5). Spring grown accessions produced 192–772 fewer seeds per flower than those in agrofabric exclosures (all *P* < 0.001, Appendix S1: Table S6, Figure S5B). In summer, most (10/12) accessions in control structures produced fewer seeds compared to those in agrofabric exclosures (297–902 fewer seeds per flower, *P* < 0.001, Appendix S1: Table S6, Figure S5B). Most plants in control structures that produced seeds in summer were coastal perennials from ocean‐exposed localities (Appendix S1: Figure S7). In the control structures, annuals and the inland perennial population did not differ in seeds per flower between seasons, largely because they produced almost no seeds (*P* ≥ 0.1, Appendix S1: Table S6, Figure S5B). In contrast, in the control structures, coastal perennials from ocean exposed and protected localities produced 22–513 fewer seeds per flower in spring compared to summer (*P* ≤ 0.03, Appendix S1: Table S6, Figure S5B). Almost all (11/12) accessions in the agrofabric exclosures did not significantly differ between seasons in seeds per flower (*P* ≥ 0.09, except one ocean‐protected coastal perennial *P* = 0.03, Appendix S1: Table S6, Figure S5B).

## DISCUSSION

The timing of flowering is a key life‐history transition that affects plant fitness. The optimal timing of reproduction depends on the costs and benefits of continued growth versus reproduction. These costs and benefits vary depending on an organism's environment, which often varies seasonally. Although much is known about the role of temperature and precipitation in driving variation in phenology, less is known about the role of hyperlocal factors like wind and oceanic salt spray. Inland annual yellow monkeyflower populations flower in early spring to avoid the summer drought, whereas coastal perennial populations inhabit seeps where moisture is available year‐round and flower in the summer (Hall and Willis, 2006; Lowry and Willis, 2010). Experimentally inducing spring flowering typical of inland populations at a coastal site resulted in high rates of necrosis, low flower production, and low seed set in all perennials and annuals spanning a coastal‐to‐inland gradient. In contrast, experimentally inducing late‐summer flowering typical of coastal populations at the same coastal site resulted in lower rates of necrosis, higher flower production, and higher seed set compared to plants exposed to the ocean in spring. Almost all individuals that produced seed when exposed to the ocean in control structures were coastal perennials from ocean‐exposed localities, consistent with prior studies demonstrating that these populations are locally adapted (Hall and Willis, 2006; Lowry et al., 2008; Popovic and Lowry, 2020). In both seasons, individuals protected from wind and salt spray with agrofabric had almost no necrosis (Figure 2) and produced more flowers and seeds than individuals exposed to the ocean in control structures (Figure 3). The benefit of protection was greater in spring than in summer (Appendix S1: Figure S2). Collectively, our results suggest that escape from tissue death is a major benefit of summer flowering in coastal populations.

Plants inhabiting coastal environments experience multiple aboveground stressors, including wind and resultant salt spray and sand abrasion (Du and Hesp, 2020). Aboveground meristems that produce new leaves or flowers and fruits are especially sensitive to these abiotic stressors (Boyce, 1954). When meristems are limiting, selection should favor strategies to either escape or tolerate recurrent meristem damage (Geber, 1990; Wise and Abrahamson, 2008). Ocean‐exposed coastal perennial monkeyflower populations likely use multiple strategies to protect their meristems from wind and salt spray, including keeping their meristems low to the ground via prostrate growth; salt spray intensity decreases with proximity to the ground (Martin, 1959; Barbour, 1978; Griffiths, 2006; Zambiasi and Lowry, 2024). Yet these strategies were insufficient to rescue spring fecundity in the populations we examined, suggesting that ocean‐exposed coastal perennial populations are constrained to reproducing during relatively still periods of the year.

In prior studies, heritable differences in life history and flowering time between coastal perennial and inland annual ecotypes were hypothesized to be primarily driven by differences in growing season length and seasonal water availability between coastal and inland environments. When transplanted in inland environments, coastal perennial ecotypes did not survive to flower before the onset of summer drought (Lowry et al., 2008; Lowry and Willis, 2010; Popovic and Lowry, 2020), and selection favored early flowering in recombinant hybrids segregating for coastal perennial and inland annual traits (Hall and Willis, 2006). When coastal perennials, inland annuals and recombinant hybrids between them were planted in coastal environments in late spring (May, notably after the windy spring season), selection favored intermediate flowering, later in the summer than in montane inland environments (Hall and Willis, 2006). The primary interpretation of this result related selection to allocation trade‐offs—plants that delay flowering can accumulate more vegetative growth for subsequent flowering throughout the longer growing seasons. While differences in growing season length are clearly important, transplant studies initiated earlier in the winter suggested that additional factors operating at different times could contribute to selection for later flowering in coastal environments. Specifically, inland annuals transplanted into coastal environments in late winter–early spring (January–March) died quickly and exhibited tissue necrosis (Lowry et al., 2008; Lowry and Willis, 2010; Popovic and Lowry, 2020; Toll et al., 2026), but annuals transplanted in June had much higher survival (Hall and Willis, 2006).

In both cohorts, all perennial accessions produced stolons and had visibly larger rosette areas, thicker stems, and larger flowers than annual accessions. As expected from prior studies (Zambiasi and Lowry, 2024), inland and protected coastal perennials were taller than ocean‐exposed coastal perennials in the spring cohort. However, over half of the accessions included in our study were taller in spring compared to summer before being moved outdoors, despite our efforts to keep growing conditions similar across seasons. While this difference in initial height may have accelerated the timing of inflorescence tissue necrosis in spring relative to summer (Appendix S1: Figure S2), we do not think it can fully explain seasonal differences in tissue necrosis and fecundity for multiple reasons. First, by the end of the summer cohort individuals were as tall or taller than the initial heights we observed in the spring cohort (Appendix S2: Figure S2), and yet the severity of tissue necrosis on both inflorescences and basal leaves was lower in the summer cohort. Furthermore, plants exposed to the ocean in control structures had uniformly higher tissue damage and lower flower production in spring compared to summer, regardless of life history, locality, or initial height. Lastly, our initial analysis of the change in living tissue height (a proxy for tissue necrosis severity) was robust to variation in initial height across season—when we incorporated covariates in our model to account for initial height differences or subset our data to only compare plants in the same height range across seasons, all plants decreased in height in spring and most increased in height in summer.

In our study region, coastal Northern California, wind speeds vary seasonally and are generally highest during upwelling (April–June), weakest from July to September, and highly variable during frequent storms from December through February (García‐Reyes and Largier, 2012). Heavy winter precipitation causes rapid leaching of salt to deeper layers of sand, ameliorating the toxic effects of soil salinity (Du and Hesp, 2020). While our study focused on temporal variation at an ocean‐exposed shoreline, perennial monkeyflowers also inhabit areas near the coast that are protected from the ocean. Protected populations can flower during the windy spring season (K. Toll, personal observations), potentially because their reproductive structures are not recurrently damaged. A prior common garden study found that ocean‐protected perennial populations are taller than nearby ocean‐exposed populations, but did not find differences in flowering time, suggesting that field phenology differences may be due to plasticity (Zambiasi and Lowry, 2024). Nevertheless, height divergence may be facilitated by assortative mating within protected and exposed forms (Aston and Bradshaw, 1966; Daehler et al., 1999; Foster et al., 2007; James et al., 2023; Itoh et al., 2024).

Removing wind and salt spray with exclosures dramatically decreased necrosis and increased fecundity relative to open structures that controlled for agrofabric shading and moisture collection. We were unable to directly separate these two factors, since high winds are ultimately the cause of salt deposition. These factors likely operate interactively because salt spray penetration increases when plant tissues are abraded by wind‐blown sand (Du and Hesp, 2020). However, we have only observed apical necrosis in natural populations of coastal monkeyflowers directly exposed to the ocean and not in nearby protected populations (Appendix S1: Figure S8), consistent with observations of shoot injury to woody plants near the ocean but not in nearby freshwater estuaries where wind speeds are similar (Wells and Shunk, 1938). Additionally, we have only observed aerial necrosis at ocean‐exposed sites in multiple reciprocal transplant experiments with yellow monkeyflowers (Lowry et al., 2008; Toll et al., 2026). Although the relative importance of wind versus salt spray in structuring plant communities has been debated (Wells and Shunk, 1938; Oosting and Billings, 1942; Boyce, 1954), the general consensus is that both factors are important in determining the distribution, abundance, and phenotypes of organisms in coastal environments (Du and Hesp, 2020). Our study adds to a growing number of recent studies directly testing the ecological significance of traits underlying adaptation to coastal environments (Wilkinson et al., 2021; Itoh et al., 2024).

## AUTHOR CONTRIBUTIONS

K.T.: methodology, investigation, formal analysis, data curation, writing original draft, review and editing, visualization. C.C.: investigation. L.T.: investigation. D.B.L.: conceptualization, methodology, review and editing, resources, supervision, funding acquisition.

## Supporting information


**Appendix S1:** Close‐up of Figure 1A inset, photographs of the experiment and natural populations, tables and plots of raw data, and tables of pairwise comparisons of analyses.

**Appendix S2:** Supplementary analysis of the change in living tissue height, a proxy for tissue necrosis, over each seasonal cohort with additional covariates (initial plant height and a height by season interaction) and using a filtered data set with only individuals that overlapped in initial height across cohorts.

## Data Availability

Raw data, metadata, and an R script are deposited in Figshare (https://doi.org/10.6084/m9.figshare.30058174).
