# Supplementary material for: Seasonal variation in wind speed and oceanic salt spray favors delayed reproduction in coastal yellow monkeyflowers
Source: Am J Bot. 2026 Aug 4;113(8):e70249. doi: 10.1002/ajb2.70249 (PMC13496057; doi:10.1002/ajb2.70249)
Supplement: Supplementary file 1 [file AJB2-113-e70249-s001.docx]

**Appendix S2.** Supplementary analysis of the change in living tissue height, a proxy for tissue necrosis, over each seasonal cohort with additional covariates (initial plant height and a height by season interaction) and using a filtered data set with only individuals that overlapped in initial height across cohorts.

Tissue necrosis, measured as the change in living tissue height, of plants exposed to the ocean in control structures was higher in the spring cohort compared to the summer cohort. Since plants were initially taller in spring than in summer, we wanted to understand how sensitive our inferences were to differences in initial plant height across seasons. Therefore, we modified the analysis the change in living tissue height presented in the main text in two ways, by (1) adding initial plant height and a height by season interaction as covariates, and (2) analyzing a data set filtered to only include individuals within each family that overlapped in initial height across seasonal cohorts (taller than the shortest plants in spring and shorter than the tallest plants in summer). In the first model, the predictor variables were accession, season, treatment, all the two-way interactions, and the three way interaction, plus height and a season by height interaction as fixed factors, and flat nested within block as random effects. In the second model, the predictor variables were identical to the model presented in the main text (accession, season, treatment, all the two-way interactions, and the three way interaction as fixed effects; flat nested within block as random effects).

In the model including the initial height covariates, necrosis, estimated by change in living tissue height, was significantly associated with all the predictor variables except for accession (Appendix S2: Table S1, all *P* < 0.03). In spring, plants exposed to the ocean in control structures significantly decreased in living tissue height (Appendix S2: Figure S1a). In summer, most plants in the control structures increased in height over time (Appendix S2: Figure S1a), but not as much as in the agrofabric exclosures (all summer control vs exclosure *P* ≤ 0.02 except for one exposed coastal perennial accession [OPB], Appendix S2: Table S2). In both seasons, plants in the agrofabric exclosures increased in height (Appendix S2: Figure S1a), and the change in height significantly differed between agrofabric exclosures and controls (all spring control vs exclosure *P* < 0.001, Appendix S2: Table S2).

In a model only including individuals within each accession that overlapped in initial height across seasons, the change in living tissue height was significantly associated with all the predictor variables (Appendix S2: Table S1, all *P* < 0.04). One of the inland annual accessions, CAV, was dropped from the analysis because few individuals overlapped in initial height across seasons. In spring, plants exposed to the ocean in control structures significantly decreased in living tissue height (Appendix S2: Figure S1b). In summer, most plants in the control structures increased in height over time (Appendix S2: Figure S1b), but not as much as in the agrofabric exclosures (all summer control vs exclosure *P* < 0.001 except for two exposed coastal perennial accessions [HEC & OPB], Appendix S2: Table S3). In both seasons, plants in the agrofabric exclosures increased in height (Appendix S2: Figure S1b), and the change in height significantly differed between agrofabric exclosures and controls (all spring control vs exclosure *P* < 0.001, Appendix S2: Table S3).

**Figure S1.** Individual height differences (final minus initial height) (background points) and predictions from a linear mixed model (black outlined points; mean and 95% confidence intervals) of the change in living tissue height over time using a model with initial height as a covariate (a) or a filtered data set of individuals within each accession that overlapped in initial height.

**Figure S2.** Histograms of initial height (in millimeters) for each accession at the beginning of each seasonal cohort (Initial [0 days]) and on the final day of each cohort (Final [21 days]). Spring heights are plotted in yellow, summer heights in orange.

**Table S1.** Analysis of deviance tables (Type III Wald *χ*^2^ tests) for models of the change in living tissue height. The covariate model includes height and a season by height interaction, the filtered model only includes individuals from each accession that overlapped in initial height across seasons.

| **Model** | **Factor** | ***χ*^2^** | **df** | ***P*** |
| --- | --- | --- | --- | --- |
| Covariate | (Intercept) | 0.92 | 1 | 0.3374 |
|  | Accession | 14.801 | 11 | **0.1918** |
|  | Season | 13.08 | 1 | **0.0003** |
|  | Treatment | 35.848 | 1 | **2.13E-09** |
|  | Height | 383.786 | 1 | **<2.2e-16** |
|  | Accession:Season | 45.293 | 11 | **4.31E-06** |
|  | Accession:Treatment | 340.724 | 11 | **<2.2e-16** |
|  | Season:Treatment | 4.897 | 1 | **0.0269** |
|  | Season:Height | 14.657 | 1 | **0.0001** |
|  | Accession:Season:Treatment | 60.275 | 11 | **8.24E-09** |
| Filtered | (Intercept) | 15.8772 | 1 | **6.76E-05** |
|  | Accession | 509.671 | 10 | **<2.2e-16** |
|  | Season | 23.4479 | 1 | **1.28E-06** |
|  | Treatment | 28.365 | 1 | **1.01E-07** |
|  | Accession:Season | 31.9035 | 10 | **0.0004** |
|  | Accession:Treatment | 225.5274 | 10 | **<2.2e-16** |
|  | Season:Treatment | 4.0743 | 1 | **0.0435** |
|  | Accession:Season:Treatment | 30.7745 | 10 | **0.0006** |

**Table S2.** Pairwise comparisons of the change in living tissue height (initial minus final height), a measure of apical necrosis. Comparisons were made from a linear mixed model consisting of accession, season, exclosure treatment, all two-way interactions, the three-way interaction, initial plant height, and a height by season interaction as fixed factors, and flat nested within block as random factors. *P*-values were adjusted using the Tukey method within each accession.

| **Accession** | **Contrast** | **Estimate** | **SE** | **df** | ***z*** | ***P*** |
| --- | --- | --- | --- | --- | --- | --- |
| HEC | Spring: Control vs Exclosure | -89.299 | 14.915 | Inf | -5.987 | 1.28E-08 |
|  | Control: Spring vs Summer | -78.191 | 13.897 | Inf | -5.627 | 1.10E-07 |
|  | Control Spring vs Exclosure Summer | -120.815 | 15.364 | Inf | -7.863 | 8.12E-14 |
|  | Exclosure Spring vs Control Summer | 11.108 | 15.386 | Inf | 0.722 | 0.888 |
|  | Exclosure: Spring vs Summer | -31.516 | 16.723 | Inf | -1.885 | 0.235 |
|  | Summer: Control vs Exclosure | -42.624 | 14.916 | Inf | -2.858 | 0.022 |
| OPB | Spring: Control vs Exclosure | -91.828 | 14.924 | Inf | -6.153 | 4.56E-09 |
|  | Control: Spring vs Summer | -72.699 | 14.08 | Inf | -5.163 | 1.45E-06 |
|  | Control Spring vs Exclosure Summer | -99.146 | 15.527 | Inf | -6.385 | 1.03E-09 |
|  | Exclosure Spring vs Control Summer | 19.129 | 15.497 | Inf | 1.234 | 0.605 |
|  | Exclosure: Spring vs Summer | -7.319 | 16.818 | Inf | -0.435 | 0.972 |
|  | Summer: Control vs Exclosure | -26.448 | 14.919 | Inf | -1.773 | 0.287 |
| SWB | Spring: Control vs Exclosure | -214.162 | 14.914 | Inf | -14.359 | 0 |
|  | Control: Spring vs Summer | -141.63 | 13.373 | Inf | -10.591 | 3.83E-14 |
|  | Control Spring vs Exclosure Summer | -228.333 | 14.984 | Inf | -15.238 | 0 |
|  | Exclosure Spring vs Control Summer | 72.532 | 14.944 | Inf | 4.854 | 7.22E-06 |
|  | Exclosure: Spring vs Summer | -14.171 | 16.404 | Inf | -0.864 | 0.823 |
|  | Summer: Control vs Exclosure | -86.703 | 14.929 | Inf | -5.808 | 3.80E-08 |
| BMR | Spring: Control vs Exclosure | -144.155 | 14.915 | Inf | -9.665 | 2.81E-14 |
|  | Control: Spring vs Summer | -109.775 | 13.784 | Inf | -7.964 | 6.52E-14 |
|  | Control Spring vs Exclosure Summer | -181.047 | 15.254 | Inf | -11.868 | 0 |
|  | Exclosure Spring vs Control Summer | 34.38 | 15.323 | Inf | 2.244 | 0.112 |
|  | Exclosure: Spring vs Summer | -36.892 | 16.658 | Inf | -2.215 | 0.119 |
|  | Summer: Control vs Exclosure | -71.272 | 14.917 | Inf | -4.778 | 1.05E-05 |
| LMC | Spring: Control vs Exclosure | -278.091 | 14.935 | Inf | -18.621 | 0 |
|  | Control: Spring vs Summer | -120.252 | 14.178 | Inf | -8.482 | 3.82E-14 |
|  | Control Spring vs Exclosure Summer | -255.116 | 15.718 | Inf | -16.231 | 0 |
|  | Exclosure Spring vs Control Summer | 157.839 | 15.884 | Inf | 9.937 | 3.49E-14 |
|  | Exclosure: Spring vs Summer | 22.975 | 17.272 | Inf | 1.33 | 0.544 |
|  | Summer: Control vs Exclosure | -134.864 | 14.916 | Inf | -9.042 | 5.40E-14 |
| PPW | Spring: Control vs Exclosure | -294.783 | 14.96 | Inf | -19.705 | 0 |
|  | Control: Spring vs Summer | -133.546 | 14.095 | Inf | -9.475 | 3.42E-14 |
|  | Control Spring vs Exclosure Summer | -272.286 | 15.596 | Inf | -17.459 | 0 |
|  | Exclosure Spring vs Control Summer | 161.237 | 15.764 | Inf | 10.228 | 3.41E-14 |
|  | Exclosure: Spring vs Summer | 22.497 | 17.119 | Inf | 1.314 | 0.554 |
|  | Summer: Control vs Exclosure | -138.74 | 14.915 | Inf | -9.302 | 4.11E-14 |
| CAV | Spring: Control vs Exclosure | -281.656 | 14.916 | Inf | -18.883 | 0 |
|  | Control: Spring vs Summer | -116.083 | 14.703 | Inf | -7.895 | 7.47E-14 |
|  | Control Spring vs Exclosure Summer | -271.931 | 16.374 | Inf | -16.607 | 0 |
|  | Exclosure Spring vs Control Summer | 165.573 | 16.111 | Inf | 10.277 | 4.36E-14 |
|  | Exclosure: Spring vs Summer | 9.725 | 17.644 | Inf | 0.551 | 0.946 |
|  | Summer: Control vs Exclosure | -155.848 | 14.946 | Inf | -10.427 | 4.13E-14 |
| MOR | Spring: Control vs Exclosure | -266.133 | 14.938 | Inf | -17.816 | 0 |
|  | Control: Spring vs Summer | -89.809 | 14.812 | Inf | -6.063 | 7.99E-09 |
|  | Control Spring vs Exclosure Summer | -303.334 | 16.264 | Inf | -18.65 | 0 |
|  | Exclosure Spring vs Control Summer | 176.324 | 16.546 | Inf | 10.657 | 3.65E-14 |
|  | Exclosure: Spring vs Summer | -37.201 | 17.857 | Inf | -2.083 | 0.159 |
|  | Summer: Control vs Exclosure | -213.525 | 14.914 | Inf | -14.317 | 0 |
| AJEN | Spring: Control vs Exclosure | -239.445 | 14.915 | Inf | -16.054 | 0 |
|  | Control: Spring vs Summer | -123.486 | 13.725 | Inf | -8.997 | 2.63E-14 |
|  | Control Spring vs Exclosure Summer | -280.195 | 15.328 | Inf | -18.28 | 0 |
|  | Exclosure Spring vs Control Summer | 115.96 | 15.226 | Inf | 7.616 | 1.82E-13 |
|  | Exclosure: Spring vs Summer | -40.749 | 16.686 | Inf | -2.442 | 0.069 |
|  | Summer: Control vs Exclosure | -156.709 | 14.921 | Inf | -10.503 | 4.16E-14 |
| SAL | Spring: Control vs Exclosure | -250.539 | 14.918 | Inf | -16.795 | 0 |
|  | Control: Spring vs Summer | -127.503 | 14.842 | Inf | -8.59 | 3.53E-14 |
|  | Control Spring vs Exclosure Summer | -274.754 | 15.941 | Inf | -17.236 | 0 |
|  | Exclosure Spring vs Control Summer | 123.035 | 16.325 | Inf | 7.537 | 3.15E-13 |
|  | Exclosure: Spring vs Summer | -24.215 | 17.33 | Inf | -1.397 | 0.501 |
|  | Summer: Control vs Exclosure | -147.251 | 14.947 | Inf | -9.851 | 3.86E-14 |
| JEN | Spring: Control vs Exclosure | -243.803 | 14.92 | Inf | -16.341 | 0 |
|  | Control: Spring vs Summer | -110.232 | 13.745 | Inf | -8.02 | 5.94E-14 |
|  | Control Spring vs Exclosure Summer | -174.342 | 15.311 | Inf | -11.387 | 0 |
|  | Exclosure Spring vs Control Summer | 133.571 | 15.347 | Inf | 8.703 | 3.26E-14 |
|  | Exclosure: Spring vs Summer | 69.461 | 16.764 | Inf | 4.143 | 0.0002 |
|  | Summer: Control vs Exclosure | -64.111 | 14.915 | Inf | -4.298 | 0.0001 |
| GUL | Spring: Control vs Exclosure | -219.922 | 14.925 | Inf | -14.735 | 0 |
|  | Control: Spring vs Summer | -145.436 | 14.009 | Inf | -10.382 | 3.51E-14 |
|  | Control Spring vs Exclosure Summer | -253.553 | 15.396 | Inf | -16.469 | 0 |
|  | Exclosure Spring vs Control Summer | 74.486 | 15.494 | Inf | 4.807 | 9.10E-06 |
|  | Exclosure: Spring vs Summer | -33.631 | 16.757 | Inf | -2.007 | 0.185 |
|  | Summer: Control vs Exclosure | -108.117 | 14.921 | Inf | -7.246 | 2.60E-12 |

**Table S3.** Pairwise comparisons of the change in living tissue height (initial minus final height), a measure of apical necrosis, on plants with overlapping initial heights within each accession across seasons. Comparisons were made from a linear mixed model consisting of accession, season, exclosure treatment, all two-way interactions, and the three-way interaction as fixed factors, and flat nested within block as random factors. *P*-values were adjusted using the Tukey method within each accession.

| **Accession** | **contrast** | **Estimate** | **SE** | **df** | | ***z*** | | ***P*** | |
| --- | --- | --- | --- | --- | --- | --- | --- | --- | --- |
| HEC | Spring: Control vs Exclosure | -84.714 | 15.906 | | Inf | | -5.326 | | 6.01E-07 |
|  | Control: Spring vs Summer | -68.467 | 14.139 | | Inf | | -4.842 | | 7.64E-06 |
|  | Control Spring vs Exclosure Summer | -107.883 | 15.907 | | Inf | | -6.782 | | 7.10E-11 |
|  | Exclosure Spring vs Control Summer | 16.247 | 15.829 | | Inf | | 1.026 | | 0.734 |
|  | Exclosure: Spring vs Summer | -23.168 | 17.427 | | Inf | | -1.329 | | 0.544 |
|  | Summer: Control vs Exclosure | -39.415 | 15.831 | | Inf | | -2.49 | | 0.061 |
| OPB | Spring: Control vs Exclosure | -105.911 | 18.617 | | Inf | | -5.689 | | 7.65E-08 |
|  | Control: Spring vs Summer | -88.288 | 18.109 | | Inf | | -4.875 | | 6.47E-06 |
|  | Control Spring vs Exclosure Summer | -111.03 | 21.447 | | Inf | | -5.177 | | 1.35E-06 |
|  | Exclosure Spring vs Control Summer | 17.623 | 19.189 | | Inf | | 0.918 | | 0.795 |
|  | Exclosure: Spring vs Summer | -5.12 | 22.364 | | Inf | | -0.229 | | 0.996 |
|  | Summer: Control vs Exclosure | -22.742 | 21.924 | | Inf | | -1.037 | | 0.727 |
| SWB | Spring: Control vs Exclosure | -216.568 | 16.025 | | Inf | | -13.514 | | 0 |
|  | Control: Spring vs Summer | -121.733 | 14.053 | | Inf | | -8.663 | | 3.36E-14 |
|  | Control Spring vs Exclosure Summer | -208.582 | 15.83 | | Inf | | -13.177 | | 0 |
|  | Exclosure Spring vs Control Summer | 94.834 | 16.027 | | Inf | | 5.917 | | 1.96E-08 |
|  | Exclosure: Spring vs Summer | 7.986 | 17.604 | | Inf | | 0.454 | | 0.969 |
|  | Summer: Control vs Exclosure | -86.849 | 15.83 | | Inf | | -5.486 | | 2.46E-07 |
| BMR | Spring: Control vs Exclosure | -149.768 | 15.584 | | Inf | | -9.611 | | 2.98E-14 |
|  | Control: Spring vs Summer | -106.921 | 14.534 | | Inf | | -7.357 | | 1.16E-12 |
|  | Control Spring vs Exclosure Summer | -172.923 | 16.827 | | Inf | | -10.277 | | 4.37E-14 |
|  | Exclosure Spring vs Control Summer | 42.847 | 15.867 | | Inf | | 2.7 | | 0.035 |
|  | Exclosure: Spring vs Summer | -23.156 | 17.99 | | Inf | | -1.287 | | 0.571 |
|  | Summer: Control vs Exclosure | -66.003 | 17.088 | | Inf | | -3.863 | | 6.49E-04 |
| LMC | Spring: Control vs Exclosure | -226.793 | 20.073 | | Inf | | -11.298 | | 5.33E-15 |
|  | Control: Spring vs Summer | -122.826 | 20.262 | | Inf | | -6.062 | | 8.07E-09 |
|  | Control Spring vs Exclosure Summer | -309.141 | 20.091 | | Inf | | -15.387 | | 0 |
|  | Exclosure Spring vs Control Summer | 103.967 | 22.155 | | Inf | | 4.693 | | 1.60E-05 |
|  | Exclosure: Spring vs Summer | -82.348 | 21.998 | | Inf | | -3.743 | | 0.001 |
|  | Summer: Control vs Exclosure | -186.315 | 22.167 | | Inf | | -8.405 | | 4.10E-14 |
| PPW | Spring: Control vs Exclosure | -354.52 | 26.544 | | Inf | | -13.356 | | 0 |
|  | Control: Spring vs Summer | -119.143 | 22.279 | | Inf | | -5.348 | | 5.32E-07 |
|  | Control Spring vs Exclosure Summer | -322.591 | 24.398 | | Inf | | -13.222 | | 0 |
|  | Exclosure Spring vs Control Summer | 235.377 | 29.613 | | Inf | | 7.948 | | 6.66E-14 |
|  | Exclosure: Spring vs Summer | 31.929 | 31.238 | | Inf | | 1.022 | | 0.736 |
|  | Summer: Control vs Exclosure | -203.448 | 27.674 | | Inf | | -7.352 | | 1.20E-12 |
| MOR | Spring: Control vs Exclosure | -255.592 | 16.9 | | Inf | | -15.124 | | 0 |
|  | Control: Spring vs Summer | -122.029 | 15.507 | | Inf | | -7.869 | | 7.96E-14 |
|  | Control Spring vs Exclosure Summer | -340.909 | 16.457 | | Inf | | -20.715 | | 0 |
|  | Exclosure Spring vs Control Summer | 133.562 | 17.314 | | Inf | | 7.714 | | 9.65E-14 |
|  | Exclosure: Spring vs Summer | -85.318 | 18.171 | | Inf | | -4.695 | | 0 |
|  | Summer: Control vs Exclosure | -218.88 | 16.884 | | Inf | | -12.964 | | 0 |
| AJEN | Spring: Control vs Exclosure | -237.505 | 31.839 | | Inf | | -7.459 | | 5.47E-13 |
|  | Control: Spring vs Summer | -106.009 | 20.397 | | Inf | | -5.197 | | 1.21E-06 |
|  | Control Spring vs Exclosure Summer | -284.534 | 27.847 | | Inf | | -10.218 | | 3.46E-14 |
|  | Exclosure Spring vs Control Summer | 131.496 | 31.416 | | Inf | | 4.186 | | 1.67E-04 |
|  | Exclosure: Spring vs Summer | -47.029 | 36.68 | | Inf | | -1.282 | | 0.574 |
|  | Summer: Control vs Exclosure | -178.525 | 27.28 | | Inf | | -6.544 | | 3.59E-10 |
| SAL | Spring: Control vs Exclosure | -244.898 | 15.693 | | Inf | | -15.605 | | 0 |
|  | Control: Spring vs Summer | -143.815 | 14.728 | | Inf | | -9.765 | | 4.37E-14 |
|  | Control Spring vs Exclosure Summer | -272.062 | 15.52 | | Inf | | -17.53 | | 0 |
|  | Exclosure Spring vs Control Summer | 101.082 | 16.56 | | Inf | | 6.104 | | 6.20E-09 |
|  | Exclosure: Spring vs Summer | -27.164 | 17.27 | | Inf | | -1.573 | | 0.394 |
|  | Summer: Control vs Exclosure | -128.246 | 16.397 | | Inf | | -7.821 | | 5.40E-14 |
| JEN | Spring: Control vs Exclosure | -225.494 | 16.169 | | Inf | | -13.946 | | 0 |
|  | Control: Spring vs Summer | -137.644 | 18.891 | | Inf | | -7.286 | | 1.94E-12 |
|  | Control Spring vs Exclosure Summer | -245.327 | 20.313 | | Inf | | -12.077 | | 0 |
|  | Exclosure Spring vs Control Summer | 87.85 | 19.97 | | Inf | | 4.399 | | 6.42E-05 |
|  | Exclosure: Spring vs Summer | -19.833 | 21.319 | | Inf | | -0.93 | | 0.7886 |
|  | Summer: Control vs Exclosure | -107.683 | 23.454 | | Inf | | -4.591 | | 0 |
| GUL | Spring: Control vs Exclosure | -223.367 | 19.33 | | Inf | | -11.555 | | 0 |
|  | Control: Spring vs Summer | -130.134 | 16.56 | | Inf | | -7.858 | | 8.24E-14 |
|  | Control Spring vs Exclosure Summer | -251.043 | 17.596 | | Inf | | -14.267 | | 0 |
|  | Exclosure Spring vs Control Summer | 93.233 | 18.551 | | Inf | | 5.026 | | 2.99E-06 |
|  | Exclosure: Spring vs Summer | -27.676 | 19.48 | | Inf | | -1.421 | | 0.486 |
|  | Summer: Control vs Exclosure | -120.909 | 16.733 | | Inf | | -7.226 | | 3.02E-12 |
