# Supplementary material for: Seasonal variation in wind speed and oceanic salt spray favors delayed reproduction in coastal yellow monkeyflowers
Source: Am J Bot. 2026 Aug 4;113(8):e70249. doi: 10.1002/ajb2.70249 (PMC13496057; doi:10.1002/ajb2.70249)
Supplement: Supplementary file 2 [file AJB2-113-e70249-s002.docx]

**Appendix S1.** Close-up of Figure 1A inset, photographs of the experiment and natural populations, tables and plots of raw data, and tables of pairwise comparisons of analyses.

**Figure S1**. Close-up of Figure 1A inset: map of collection localities of ocean-exposed coastal perennial (dark blue circles), inland annual (red squares), ocean-protected coastal annual (orange triangle), and ocean-protected coastal perennial (light blue triangles) accessions used in the experiment.

**Figure S2**. Plants in the spring cohort had more severe necrosis, and agrofabric exclosures effectively protected plants from necrosis. Necrosis was assessed in three categories of severity (no [0], partial [1], complete [2]) before plants were moved outside from the greenhouse at the Bodega Marine Reserve (spring: 4/28/23; summer: 7/28/23), and during three weekly censuses while plants were outdoors in control (ocean-exposed) structures or agrofabric-covered exclosures (spring: 5/5/23, 5/12/23, 5/19/23; summer: 8/4/23, 8/11/23, 8/18/23).

**Figure S3.** Photographs of necrosis in (a) spring plants one week after ocean exposure and (b) summer plants three weeks after exposure. (a) depicts a local flowering exposed coastal perennial accession in the foreground next to severely necrotic annuals.

**Figure S4.** Photographs of a control and exclosure block in the spring cohort the day plants were first placed outside (initial) and a week later. Spring plants exposed to the ocean had substantial necrosis after 1 week; agrofabric-protected plants were largely undamaged.

**Figure S5**. Plants produced fewer flowers (A) and seeds per flower (B) in the spring cohort compared to the summer cohort when exposed to the ocean, plants protected by agrofabric exclosures produced more flowers and seeds than those exposed to the ocean, and the benefit of this protection was greater in spring when wind speeds and salt deposition was higher. (A) Plots of post hoc pairwise comparison estimates from a mixed model (black outlined points; mean and 95% confidence intervals). Flower production was the response variable; accession, exclosure treatment, season, all two-way interactions, and the three-way interaction were fixed predictor variables, and block nested within flat was a random effect. (B) The pairwise comparisons for seed production were from a mixed model model that included the same predictor variables as flower production except for the three-way accession by season by exclosure treatment interaction. Pairwise comparisons were performed within each accession for every combination of exclosure treatment and season, but only the relevant within season between exclosure treatment and within exclosure treatment between season comparisons are plotted (full comparisons in Tables S5 and S6).

**Figure S6.** Raw values (points) and boxplots of flowers produced during the 21 day exposure period by each plant in each cohort.

**Figure S7.** Raw values (points) and boxplots of seeds per hand-pollinated flower (up to 3 flowers per plant) produced by each plant in each cohort.

**Figure S8.** Photographs of necrosis of plants in natural coastal perennial populations exposed to the ocean observed on Bodega Head in May 2025.

**Table S1.** Accessions used in the experiments and coordinates of their original collection localities.

| Accession | Accession Type | Latitude | Longitude |
| --- | --- | --- | --- |
| HEC | Exposed Coastal Perennial | 44.13506 | -124.1228 |
| OPB | Exposed Coastal Perennial | 42.46401 | -124.42291 |
| SWB | Exposed Coastal Perennial | 39.03598 | -123.69046 |
| BMR | Exposed Coastal Perennial | 38.31608 | -123.06907 |
| LMC | Inland Annual | 38.86398 | -123.08391 |
| PPW | Inland Annual | 38.5755 | -122.7009 |
| CAV | Inland Annual | 38.34281 | -122.4854 |
| MOR | Inland Annual | 38.42958 | -122.94496 |
| SAL | Inland Perennial | 41.33966 | -123.38816 |
| AJEN | Protected Coastal Annual | 38.45672 | -123.11599 |
| JEN | Protected Coastal Perennial | 38.4677 | -123.12831 |
| GUL | Protected Coastal Perennial | 38.481706 | -123.13299 |

**Table S2.** Pairwise comparisons of initial plant height (measured in a greenhouse, just prior to placing plants outdoors). Comparisons were made from a linear mixed model consisting of accession, season, and an accession by season interaction as fixed factors, and flat as a random factor. Only the subset of relevant comparisons (within season across accessions, across seasons within accession) are reported from a larger Tukey post hoc contrast.

| **Accession or season** | **Contrast** | **Estimate** | **SE** | **df** | ***z*** | ***P*** |
| --- | --- | --- | --- | --- | --- | --- |
| AJEN | Spring - Summer | 100.770 | 8.854 | Inf | 11.382 | 0 |
| BMR | Spring - Summer | 17.194 | 8.857 | Inf | 1.941 | 0.96132347 |
| CAV | Spring - Summer | 194.303 | 8.858 | Inf | 21.934 | 0 |
| GUL | Spring - Summer | 57.091 | 8.854 | Inf | 6.448 | 3.11E-08 |
| HEC | Spring - Summer | 11.470 | 8.854 | Inf | 1.296 | 0.99986667 |
| JEN | Spring - Summer | 107.040 | 8.853 | Inf | 12.090 | 0 |
| LMC | Spring - Summer | 155.394 | 8.855 | Inf | 17.549 | 0 |
| MOR | Spring - Summer | 68.680 | 8.857 | Inf | 7.755 | 2.58E-12 |
| OPB | Spring - Summer | 4.729 | 8.858 | Inf | 0.534 | 1 |
| PPW | Spring - Summer | 135.206 | 8.853 | Inf | 15.272 | 0 |
| SAL | Spring - Summer | -39.909 | 8.853 | Inf | -4.508 | 0.0016031 |
| SWB | Spring - Summer | -33.990 | 8.854 | Inf | -3.839 | 0.02474828 |
| Spring | AJEN - BMR | 95.920 | 8.331 | Inf | 11.513 | 0 |
| Spring | AJEN - CAV | -58.096 | 8.336 | Inf | -6.970 | 8.76E-10 |
| Spring | AJEN - GUL | 77.618 | 8.330 | Inf | 9.318 | 2.43E-13 |
| Spring | AJEN - HEC | 105.652 | 8.298 | Inf | 12.732 | 0 |
| Spring | AJEN - JEN | -4.280 | 8.330 | Inf | -0.514 | 1 |
| Spring | AJEN - LMC | -42.958 | 8.331 | Inf | -5.156 | 6.67E-05 |
| Spring | AJEN - MOR | -80.656 | 8.331 | Inf | -9.681 | 2.94E-13 |
| Spring | AJEN - OPB | 118.079 | 8.336 | Inf | 14.166 | 0 |
| Spring | AJEN - PPW | 5.667 | 8.330 | Inf | 0.680 | 1 |
| Spring | AJEN - SAL | -14.661 | 8.314 | Inf | -1.763 | 0.98737912 |
| Spring | AJEN - SWB | 70.889 | 8.314 | Inf | 8.527 | 2.24E-13 |
| Spring | BMR - CAV | -154.017 | 8.343 | Inf | -18.460 | 0 |
| Spring | BMR - GUL | -18.302 | 8.315 | Inf | -2.201 | 0.87025536 |
| Spring | BMR - HEC | 9.731 | 8.331 | Inf | 1.168 | 0.99997779 |
| Spring | BMR - JEN | -100.200 | 8.316 | Inf | -12.049 | 0 |
| Spring | BMR - LMC | -138.879 | 8.336 | Inf | -16.661 | 0 |
| Spring | BMR - MOR | -176.577 | 8.298 | Inf | -21.280 | 0 |
| Spring | BMR - OPB | 22.159 | 8.343 | Inf | 2.656 | 0.55178704 |
| Spring | BMR - PPW | -90.253 | 8.331 | Inf | -10.833 | 2.30E-13 |
| Spring | BMR - SAL | -110.581 | 8.332 | Inf | -13.271 | 0 |
| Spring | BMR - SWB | -25.032 | 8.331 | Inf | -3.005 | 0.29091805 |
| Spring | CAV - GUL | 135.714 | 8.337 | Inf | 16.278 | 0 |
| Spring | CAV - HEC | 163.748 | 8.336 | Inf | 19.645 | 0 |
| Spring | CAV - JEN | 53.817 | 8.318 | Inf | 6.470 | 2.70E-08 |
| Spring | CAV - LMC | 15.138 | 8.315 | Inf | 1.820 | 0.98137892 |
| Spring | CAV - MOR | -22.560 | 8.343 | Inf | -2.704 | 0.51291082 |
| Spring | CAV - OPB | 176.175 | 8.298 | Inf | 21.231 | 0 |
| Spring | CAV - PPW | 63.763 | 8.335 | Inf | 7.650 | 5.69E-12 |
| Spring | CAV - SAL | 43.435 | 8.334 | Inf | 5.212 | 4.98E-05 |
| Spring | CAV - SWB | 128.985 | 8.337 | Inf | 15.472 | 0 |
| Spring | GUL - HEC | 28.034 | 8.330 | Inf | 3.365 | 0.116265 |
| Spring | GUL - JEN | -81.897 | 8.330 | Inf | -9.832 | 2.42E-13 |
| Spring | GUL - LMC | -120.576 | 8.332 | Inf | -14.472 | 0 |
| Spring | GUL - MOR | -158.274 | 8.315 | Inf | -19.036 | 0 |
| Spring | GUL - OPB | 40.461 | 8.337 | Inf | 4.853 | 0.00031288 |
| Spring | GUL - PPW | -71.951 | 8.314 | Inf | -8.654 | 2.03E-13 |
| Spring | GUL - SAL | -92.279 | 8.314 | Inf | -11.099 | 1.01E-13 |
| Spring | GUL - SWB | -6.729 | 8.330 | Inf | -0.808 | 0.99999998 |
| Spring | HEC - JEN | -109.931 | 8.330 | Inf | -13.197 | 0 |
| Spring | HEC - LMC | -148.610 | 8.331 | Inf | -17.838 | 0 |
| Spring | HEC - MOR | -186.308 | 8.331 | Inf | -22.362 | 0 |
| Spring | HEC - OPB | 12.428 | 8.336 | Inf | 1.491 | 0.99875319 |
| Spring | HEC - PPW | -99.985 | 8.330 | Inf | -12.003 | 0 |
| Spring | HEC - SAL | -120.312 | 8.314 | Inf | -14.471 | 0 |
| Spring | HEC - SWB | -34.763 | 8.314 | Inf | -4.181 | 0.0065663 |
| Spring | JEN - LMC | -38.679 | 8.330 | Inf | -4.643 | 0.00085946 |
| Spring | JEN - MOR | -76.377 | 8.316 | Inf | -9.184 | 2.80E-13 |
| Spring | JEN - OPB | 122.359 | 8.318 | Inf | 14.710 | 0 |
| Spring | JEN - PPW | 9.947 | 8.330 | Inf | 1.194 | 0.99996716 |
| Spring | JEN - SAL | -10.381 | 8.346 | Inf | -1.244 | 0.99993315 |
| Spring | JEN - SWB | 75.168 | 8.314 | Inf | 9.041 | 3.23E-13 |
| Spring | LMC - MOR | -37.698 | 8.336 | Inf | -4.522 | 0.00149947 |
| Spring | LMC - OPB | 161.038 | 8.315 | Inf | 19.367 | 0 |
| Spring | LMC - PPW | 48.625 | 8.315 | Inf | 5.848 | 1.36E-06 |
| Spring | LMC - SAL | 28.298 | 8.315 | Inf | 3.403 | 0.10413238 |
| Spring | LMC - SWB | 113.847 | 8.332 | Inf | 13.664 | 0 |
| Spring | MOR - OPB | 198.735 | 8.343 | Inf | 23.820 | 0 |
| Spring | MOR - PPW | 86.323 | 8.331 | Inf | 10.361 | 2.21E-13 |
| Spring | MOR - SAL | 65.995 | 8.332 | Inf | 7.920 | 9.89E-13 |
| Spring | MOR - SWB | 151.545 | 8.331 | Inf | 18.191 | 0 |
| Spring | OPB - PPW | -112.412 | 8.335 | Inf | -13.486 | 0 |
| Spring | OPB - SAL | -132.740 | 8.334 | Inf | -15.927 | 0 |
| Spring | OPB - SWB | -47.190 | 8.337 | Inf | -5.661 | 4.10E-06 |
| Spring | PPW - SAL | -20.328 | 8.314 | Inf | -2.445 | 0.71755968 |
| Spring | PPW - SWB | 65.222 | 8.314 | Inf | 7.845 | 1.55E-12 |
| Spring | SAL - SWB | 85.549 | 8.330 | Inf | 10.270 | 2.66E-13 |
| Summer | AJEN - BMR | 12.344 | 8.330 | Inf | 1.482 | 0.99886185 |
| Summer | AJEN - CAV | 35.437 | 8.330 | Inf | 4.254 | 0.00485024 |
| Summer | AJEN - GUL | 33.939 | 8.330 | Inf | 4.074 | 0.01011086 |
| Summer | AJEN - HEC | 16.352 | 8.298 | Inf | 1.971 | 0.95461189 |
| Summer | AJEN - JEN | 1.991 | 8.330 | Inf | 0.239 | 1 |
| Summer | AJEN - LMC | 11.665 | 8.330 | Inf | 1.400 | 0.99952547 |
| Summer | AJEN - MOR | -112.746 | 8.330 | Inf | -13.535 | 0 |
| Summer | AJEN - OPB | 22.039 | 8.330 | Inf | 2.646 | 0.56006862 |
| Summer | AJEN - PPW | 40.104 | 8.330 | Inf | 4.815 | 0.0003783 |
| Summer | AJEN - SAL | -155.339 | 8.314 | Inf | -18.684 | 0 |
| Summer | AJEN - SWB | -63.871 | 8.314 | Inf | -7.683 | 4.44E-12 |
| Summer | BMR - CAV | 23.093 | 8.330 | Inf | 2.772 | 0.45854916 |
| Summer | BMR - GUL | 21.595 | 8.314 | Inf | 2.598 | 0.59902444 |
| Summer | BMR - HEC | 4.008 | 8.330 | Inf | 0.481 | 1 |
| Summer | BMR - JEN | -10.353 | 8.314 | Inf | -1.245 | 0.99993182 |
| Summer | BMR - LMC | -0.679 | 8.330 | Inf | -0.081 | 1 |
| Summer | BMR - MOR | -125.090 | 8.298 | Inf | -15.075 | 0 |
| Summer | BMR - OPB | 9.694 | 8.330 | Inf | 1.164 | 0.99997918 |
| Summer | BMR - PPW | 27.759 | 8.330 | Inf | 3.332 | 0.12771489 |
| Summer | BMR - SAL | -167.683 | 8.330 | Inf | -20.130 | 0 |
| Summer | BMR - SWB | -76.215 | 8.330 | Inf | -9.150 | 2.91E-13 |
| Summer | CAV - GUL | -1.497 | 8.330 | Inf | -0.180 | 1 |
| Summer | CAV - HEC | -19.085 | 8.330 | Inf | -2.291 | 0.82098368 |
| Summer | CAV - JEN | -33.446 | 8.314 | Inf | -4.023 | 0.01238536 |
| Summer | CAV - LMC | -23.771 | 8.314 | Inf | -2.859 | 0.39180288 |
| Summer | CAV - MOR | -148.183 | 8.330 | Inf | -17.789 | 0 |
| Summer | CAV - OPB | -13.398 | 8.298 | Inf | -1.615 | 0.99605125 |
| Summer | CAV - PPW | 4.667 | 8.330 | Inf | 0.560 | 1 |
| Summer | CAV - SAL | -190.776 | 8.330 | Inf | -22.903 | 0 |
| Summer | CAV - SWB | -99.308 | 8.330 | Inf | -11.922 | 0 |
| Summer | GUL - HEC | -17.588 | 8.330 | Inf | -2.111 | 0.91020489 |
| Summer | GUL - JEN | -31.948 | 8.330 | Inf | -3.835 | 0.02508132 |
| Summer | GUL - LMC | -22.274 | 8.330 | Inf | -2.674 | 0.53713493 |
| Summer | GUL - MOR | -146.685 | 8.314 | Inf | -17.644 | 0 |
| Summer | GUL - OPB | -11.901 | 8.330 | Inf | -1.429 | 0.99935055 |
| Summer | GUL - PPW | 6.164 | 8.314 | Inf | 0.741 | 1 |
| Summer | GUL - SAL | -189.279 | 8.314 | Inf | -22.767 | 0 |
| Summer | GUL - SWB | -97.811 | 8.330 | Inf | -11.742 | 0 |
| Summer | HEC - JEN | -14.361 | 8.330 | Inf | -1.724 | 0.99051775 |
| Summer | HEC - LMC | -4.686 | 8.330 | Inf | -0.563 | 1 |
| Summer | HEC - MOR | -129.098 | 8.330 | Inf | -15.498 | 0 |
| Summer | HEC - OPB | 5.687 | 8.330 | Inf | 0.683 | 1 |
| Summer | HEC - PPW | 23.752 | 8.330 | Inf | 2.851 | 0.39766882 |
| Summer | HEC - SAL | -171.691 | 8.314 | Inf | -20.651 | 0 |
| Summer | HEC - SWB | -80.223 | 8.314 | Inf | -9.649 | 1.73E-13 |
| Summer | JEN - LMC | 9.675 | 8.330 | Inf | 1.161 | 0.99997992 |
| Summer | JEN - MOR | -114.737 | 8.314 | Inf | -13.801 | 0 |
| Summer | JEN - OPB | 20.048 | 8.314 | Inf | 2.411 | 0.74198823 |
| Summer | JEN - PPW | 38.113 | 8.330 | Inf | 4.575 | 0.00117686 |
| Summer | JEN - SAL | -157.330 | 8.346 | Inf | -18.851 | 0 |
| Summer | JEN - SWB | -65.862 | 8.314 | Inf | -7.922 | 9.81E-13 |
| Summer | LMC - MOR | -124.411 | 8.330 | Inf | -14.935 | 0 |
| Summer | LMC - OPB | 10.373 | 8.314 | Inf | 1.248 | 0.99992954 |
| Summer | LMC - PPW | 28.438 | 8.314 | Inf | 3.421 | 0.09896107 |
| Summer | LMC - SAL | -167.005 | 8.314 | Inf | -20.088 | 0 |
| Summer | LMC - SWB | -75.537 | 8.330 | Inf | -9.068 | 3.13E-13 |
| Summer | MOR - OPB | 134.784 | 8.330 | Inf | 16.181 | 0 |
| Summer | MOR - PPW | 152.849 | 8.330 | Inf | 18.349 | 0 |
| Summer | MOR - SAL | -42.594 | 8.330 | Inf | -5.113 | 8.36E-05 |
| Summer | MOR - SWB | 48.874 | 8.330 | Inf | 5.867 | 1.21E-06 |
| Summer | OPB - PPW | 18.065 | 8.330 | Inf | 2.169 | 0.88578692 |
| Summer | OPB - SAL | -177.378 | 8.330 | Inf | -21.294 | 0 |
| Summer | OPB - SWB | -85.910 | 8.330 | Inf | -10.314 | 2.42E-13 |
| Summer | PPW - SAL | -195.443 | 8.314 | Inf | -23.508 | 0 |
| Summer | PPW - SWB | -103.975 | 8.314 | Inf | -12.506 | 0 |
| Summer | SAL - SWB | 91.468 | 8.330 | Inf | 10.981 | 1.97E-13 |

**Table S3.** Pairwise comparisons of the change in living tissue height (initial minus final height), a measure of apical necrosis. Comparisons were made from a linear mixed model consisting of accession, season, exclosure treatment, all two-way interactions, and the three-way interaction as fixed factors, and flat nested within block as random factors. *P*-values were adjusted using the Tukey method within each accession.

| **Accession** | **Contrast** | **Estimate** | **SE** | **df** | ***z*** | ***P*** |
| --- | --- | --- | --- | --- | --- | --- |
| HEC | Spring: Control vs Exclosure | -152.347 | 10.615 | 4222 | -14.352 | 3.70E-08 |
|  | Control: Spring vs Summer | -37.861 | 9.494 | 4222 | -3.988 | 0.000394544 |
|  | Control Spring vs Exclosure Summer | -113.847 | 10.615 | 4222 | -10.725 | 3.70E-08 |
|  | Exclosure Spring vs Control Summer | 114.486 | 10.615 | 4222 | 10.785 | 3.70E-08 |
|  | Exclosure: Spring vs Summer | 38.500 | 11.628 | 4222 | 3.311 | 0.005186841 |
|  | Summer: Control vs Exclosure | -75.986 | 10.615 | 4222 | -7.158 | 3.70E-08 |
| OPB | Spring: Control vs Exclosure | -74.621 | 10.615 | 4222 | -7.030 | 3.70E-08 |
|  | Control: Spring vs Summer | -38.121 | 9.494 | 4222 | -4.015 | 0.000352102 |
|  | Control Spring vs Exclosure Summer | -80.135 | 10.615 | 4222 | -7.549 | 3.70E-08 |
|  | Exclosure Spring vs Control Summer | 36.500 | 10.615 | 4222 | 3.438 | 0.003308706 |
|  | Exclosure: Spring vs Summer | -5.514 | 11.628 | 4222 | -0.474 | 0.964765518 |
|  | Summer: Control vs Exclosure | -42.014 | 10.615 | 4222 | -3.958 | 0.000446205 |
| SWB | Spring: Control vs Exclosure | -186.208 | 10.615 | 4222 | -17.542 | 3.70E-08 |
|  | Control: Spring vs Summer | 11.519 | 9.494 | 4222 | 1.213 | 0.61846304 |
|  | Control Spring vs Exclosure Summer | -44.069 | 10.615 | 4222 | -4.152 | 0.00019722 |
|  | Exclosure Spring vs Control Summer | 197.727 | 10.615 | 4222 | 18.627 | 3.70E-08 |
|  | Exclosure: Spring vs Summer | 142.139 | 11.628 | 4222 | 12.224 | 3.70E-08 |
|  | Summer: Control vs Exclosure | -55.588 | 10.615 | 4222 | -5.237 | 1.06E-06 |
| BMR | Spring: Control vs Exclosure | -129.435 | 10.615 | 4222 | -12.193 | 3.70E-08 |
|  | Control: Spring vs Summer | -40.731 | 9.494 | 4222 | -4.290 | 0.000107502 |
|  | Control Spring vs Exclosure Summer | -109.213 | 10.615 | 4222 | -10.288 | 3.70E-08 |
|  | Exclosure Spring vs Control Summer | 88.704 | 10.615 | 4222 | 8.356 | 3.70E-08 |
|  | Exclosure: Spring vs Summer | 20.222 | 11.628 | 4222 | 1.739 | 0.303511085 |
|  | Summer: Control vs Exclosure | -68.481 | 10.615 | 4222 | -6.451 | 3.78E-08 |
| LMC | Spring: Control vs Exclosure | -51.306 | 10.615 | 4222 | -4.833 | 8.32E-06 |
|  | Control: Spring vs Summer | -30.195 | 9.494 | 4222 | -3.180 | 0.008074192 |
|  | Control Spring vs Exclosure Summer | -58.792 | 10.615 | 4222 | -5.539 | 2.31E-07 |
|  | Exclosure Spring vs Control Summer | 21.111 | 10.615 | 4222 | 1.989 | 0.192315624 |
|  | Exclosure: Spring vs Summer | -7.486 | 11.628 | 4222 | -0.644 | 0.917731717 |
|  | Summer: Control vs Exclosure | -28.597 | 10.615 | 4222 | -2.694 | 0.035684182 |
| PPW | Spring: Control vs Exclosure | -162.194 | 10.615 | 4222 | -15.280 | 3.70E-08 |
|  | Control: Spring vs Summer | -22.564 | 9.494 | 4222 | -2.377 | 0.081756765 |
|  | Control Spring vs Exclosure Summer | -54.499 | 10.615 | 4222 | -5.134 | 1.81E-06 |
|  | Exclosure Spring vs Control Summer | 139.630 | 10.615 | 4222 | 13.154 | 3.70E-08 |
|  | Exclosure: Spring vs Summer | 107.695 | 11.628 | 4222 | 9.261 | 3.70E-08 |
|  | Summer: Control vs Exclosure | -31.935 | 10.615 | 4222 | -3.008 | 0.014056293 |
| CAV | Spring: Control vs Exclosure | -179.310 | 10.615 | 4222 | -16.892 | 3.70E-08 |
|  | Control: Spring vs Summer | -14.157 | 9.494 | 4222 | -1.491 | 0.442942608 |
|  | Control Spring vs Exclosure Summer | -75.532 | 10.615 | 4222 | -7.116 | 3.70E-08 |
|  | Exclosure Spring vs Control Summer | 165.153 | 10.615 | 4222 | 15.558 | 3.70E-08 |
|  | Exclosure: Spring vs Summer | 103.778 | 11.628 | 4222 | 8.925 | 3.70E-08 |
|  | Summer: Control vs Exclosure | -61.375 | 10.615 | 4222 | -5.782 | 8.45E-08 |
| MOR | Spring: Control vs Exclosure | -180.296 | 10.615 | 4222 | -16.985 | 3.70E-08 |
|  | Control: Spring vs Summer | -72.389 | 9.494 | 4222 | -7.624 | 3.70E-08 |
|  | Control Spring vs Exclosure Summer | -173.782 | 10.615 | 4222 | -16.371 | 3.70E-08 |
|  | Exclosure Spring vs Control Summer | 107.907 | 10.615 | 4222 | 10.165 | 3.70E-08 |
|  | Exclosure: Spring vs Summer | 6.514 | 11.628 | 4222 | 0.560 | 0.943785097 |
|  | Summer: Control vs Exclosure | -101.393 | 10.615 | 4222 | -9.552 | 3.70E-08 |
| AJEN | Spring: Control vs Exclosure | -50.454 | 10.615 | 4222 | -4.753 | 1.23E-05 |
|  | Control: Spring vs Summer | -25.092 | 9.494 | 4222 | -2.643 | 0.041101817 |
|  | Control Spring vs Exclosure Summer | -41.314 | 10.615 | 4222 | -3.892 | 0.000584235 |
|  | Exclosure Spring vs Control Summer | 25.361 | 10.615 | 4222 | 2.389 | 0.07928762 |
|  | Exclosure: Spring vs Summer | 9.139 | 11.628 | 4222 | 0.786 | 0.860866586 |
|  | Summer: Control vs Exclosure | -16.222 | 10.615 | 4222 | -1.528 | 0.420612157 |
| SAL | Spring: Control vs Exclosure | -196.736 | 10.615 | 4222 | -18.534 | 3.70E-08 |
|  | Control: Spring vs Summer | -9.917 | 9.494 | 4222 | -1.045 | 0.723216565 |
|  | Control Spring vs Exclosure Summer | -71.042 | 10.615 | 4222 | -6.693 | 3.72E-08 |
|  | Exclosure Spring vs Control Summer | 186.819 | 10.615 | 4222 | 17.599 | 3.70E-08 |
|  | Exclosure: Spring vs Summer | 125.694 | 11.628 | 4222 | 10.809 | 3.70E-08 |
|  | Summer: Control vs Exclosure | -61.125 | 10.615 | 4222 | -5.758 | 9.15E-08 |
| JEN | Spring: Control vs Exclosure | -152.972 | 10.615 | 4222 | -14.411 | 3.70E-08 |
|  | Control: Spring vs Summer | -138.630 | 9.494 | 4222 | -14.601 | 3.70E-08 |
|  | Control Spring vs Exclosure Summer | -195.834 | 10.615 | 4222 | -18.449 | 3.70E-08 |
|  | Exclosure Spring vs Control Summer | 14.343 | 10.615 | 4222 | 1.351 | 0.530290895 |
|  | Exclosure: Spring vs Summer | -42.861 | 11.628 | 4222 | -3.686 | 0.001318564 |
|  | Summer: Control vs Exclosure | -57.204 | 10.615 | 4222 | -5.389 | 4.84E-07 |
| GUL | Spring: Control vs Exclosure | -123.866 | 10.615 | 4222 | -11.669 | 3.70E-08 |
|  | Control: Spring vs Summer | -105.055 | 9.494 | 4222 | -11.065 | 3.70E-08 |
|  | Control Spring vs Exclosure Summer | -153.435 | 10.615 | 4222 | -14.454 | 3.70E-08 |
|  | Exclosure Spring vs Control Summer | 18.810 | 10.615 | 4222 | 1.772 | 0.287005258 |
|  | Exclosure: Spring vs Summer | -29.569 | 11.628 | 4222 | -2.543 | 0.053714451 |
|  | Summer: Control vs Exclosure | -48.379 | 10.615 | 4222 | -4.558 | 3.16E-05 |

**Table S4.** Overall counts of leaf and inflorescence necrosis by the end of the spring and summer exposure periods.

| Season | Treatment | Leaf Necrosis | | | Inflorescence Necrosis | | |
| --- | --- | --- | --- | --- | --- | --- | --- |
|  |  | None | Partial | Complete | None | Partial | Complete |
| Spring | Control | 0 | 10 | 314 | 0 | 1 | 323 |
|  | Exclosure | 190 | 20 | 6 | 196 | 6 | 14 |
| Summer | Control | 56 | 268 | 0 | 71 | 253 | 0 |
|  | Exclosure | 216 | 0 | 0 | 216 | 0 | 0 |

**Table S5**. Pairwise comparisons of flowers produced over 21 days outdoors. Comparisons were made from a mixed model with a negative binomial error distribution consisting of accession, season, exclosure treatment, all two-way interactions, and the three-way interaction as fixed factors, and flat nested within block as random factors. *P*-values were adjusted using the Tukey method within each accession.

| **Accession** | **Contrast** | **Estimate** | **SE** | **df** | ***z*** | ***P*** |
| --- | --- | --- | --- | --- | --- | --- |
| HEC | Spring: Control vs Exclosure | -8.147 | 1.532 | Inf | -5.316 | 6.33E-07 |
|  | Control: Spring vs Summer | -6.176 | 1.134 | Inf | -5.446 | 3.09E-07 |
|  | Control Spring vs Exclosure Summer | -7.481 | 1.390 | Inf | -5.383 | 4.37E-07 |
|  | Exclosure Spring vs Control Summer | 1.971 | 1.852 | Inf | 1.064 | 0.71132193 |
|  | Exclosure: Spring vs Summer | 0.666 | 2.019 | Inf | 0.330 | 0.987614849 |
|  | Summer: Control vs Exclosure | -1.305 | 1.736 | Inf | -0.752 | 0.875968098 |
| OPB | Spring: Control vs Exclosure | -4.912 | 0.997 | Inf | -4.927 | 4.98E-06 |
|  | Control: Spring vs Summer | -5.308 | 0.873 | Inf | -6.078 | 7.30E-09 |
|  | Control Spring vs Exclosure Summer | -6.637 | 1.176 | Inf | -5.645 | 9.92E-08 |
|  | Exclosure Spring vs Control Summer | -0.397 | 1.287 | Inf | -0.308 | 0.989833006 |
|  | Exclosure: Spring vs Summer | -1.726 | 1.509 | Inf | -1.144 | 0.662181368 |
|  | Summer: Control vs Exclosure | -1.329 | 1.430 | Inf | -0.929 | 0.789058137 |
| SWB | Spring: Control vs Exclosure | -5.201 | 0.936 | Inf | -5.558 | 1.64E-07 |
|  | Control: Spring vs Summer | -6.950 | 0.974 | Inf | -7.134 | 5.86E-12 |
|  | Control Spring vs Exclosure Summer | -12.151 | 1.782 | Inf | -6.819 | 5.49E-11 |
|  | Exclosure Spring vs Control Summer | -1.749 | 1.333 | Inf | -1.312 | 0.555158244 |
|  | Exclosure: Spring vs Summer | -6.950 | 2.001 | Inf | -3.474 | 0.002886125 |
|  | Summer: Control vs Exclosure | -5.201 | 2.019 | Inf | -2.576 | 0.049027677 |
| BMR | Spring: Control vs Exclosure | -4.564 | 0.941 | Inf | -4.852 | 7.27E-06 |
|  | Control: Spring vs Summer | -6.554 | 1.078 | Inf | -6.080 | 7.20E-09 |
|  | Control Spring vs Exclosure Summer | -7.466 | 1.284 | Inf | -5.815 | 3.63E-08 |
|  | Exclosure Spring vs Control Summer | -1.990 | 1.392 | Inf | -1.430 | 0.480391334 |
|  | Exclosure: Spring vs Summer | -2.902 | 1.557 | Inf | -1.863 | 0.243977877 |
|  | Summer: Control vs Exclosure | -0.911 | 1.644 | Inf | -0.554 | 0.945400374 |
| LMC | Spring: Control vs Exclosure | -14.268 | 2.193 | Inf | -6.505 | 4.66E-10 |
|  | Control: Spring vs Summer | -4.039 | 0.660 | Inf | -6.117 | 5.72E-09 |
|  | Control Spring vs Exclosure Summer | -10.996 | 1.606 | Inf | -6.846 | 4.56E-11 |
|  | Exclosure Spring vs Control Summer | 10.229 | 2.285 | Inf | 4.476 | 4.50E-05 |
|  | Exclosure: Spring vs Summer | 3.272 | 2.714 | Inf | 1.206 | 0.623314312 |
|  | Summer: Control vs Exclosure | -6.957 | 1.730 | Inf | -4.022 | 3.36E-04 |
| PPW | Spring: Control vs Exclosure | -11.038 | 1.714 | Inf | -6.439 | 7.21E-10 |
|  | Control: Spring vs Summer | -1.463 | 0.288 | Inf | -5.071 | 2.36177E-06 |
|  | Control Spring vs Exclosure Summer | -5.772 | 0.913 | Inf | -6.321 | 1.56E-09 |
|  | Exclosure Spring vs Control Summer | 9.575 | 1.737 | Inf | 5.511 | 2.13E-07 |
|  | Exclosure: Spring vs Summer | 5.266 | 1.941 | Inf | 2.712 | 0.033786907 |
|  | Summer: Control vs Exclosure | -4.309 | 0.956 | Inf | -4.507 | 3.90E-05 |
| CAV | Spring: Control vs Exclosure | -13.861 | 2.175 | Inf | -6.373 | 1.11E-09 |
|  | Control: Spring vs Summer | -2.058 | 0.436 | Inf | -4.722 | 1.39E-05 |
|  | Control Spring vs Exclosure Summer | -5.699 | 0.929 | Inf | -6.131 | 5.23E-09 |
|  | Exclosure Spring vs Control Summer | 11.802 | 2.215 | Inf | 5.329 | 5.90E-07 |
|  | Exclosure: Spring vs Summer | 8.162 | 2.362 | Inf | 3.456 | 0.003080277 |
|  | Summer: Control vs Exclosure | -3.640 | 1.019 | Inf | -3.573 | 0.002000462 |
| MOR | Spring: Control vs Exclosure | -14.176 | 2.020 | Inf | -7.017 | 1.36E-11 |
|  | Control: Spring vs Summer | -4.573 | 0.700 | Inf | -6.532 | 3.88E-10 |
|  | Control Spring vs Exclosure Summer | -28.824 | 3.776 | Inf | -7.634 | 1.61E-13 |
|  | Exclosure Spring vs Control Summer | 9.603 | 2.137 | Inf | 4.494 | 4.13E-05 |
|  | Exclosure: Spring vs Summer | -14.649 | 4.281 | Inf | -3.421 | 0.003485861 |
|  | Summer: Control vs Exclosure | -24.252 | 3.839 | Inf | -6.316 | 1.61E-09 |
| AJEN | Spring: Control vs Exclosure | -7.956 | 1.284 | Inf | -6.195 | 3.50E-09 |
|  | Control: Spring vs Summer | -3.013 | 0.528 | Inf | -5.703 | 7.07E-08 |
|  | Control Spring vs Exclosure Summer | -6.697 | 1.074 | Inf | -6.238 | 2.67E-09 |
|  | Exclosure Spring vs Control Summer | 4.942 | 1.379 | Inf | 3.583 | 0.00192531 |
|  | Exclosure: Spring vs Summer | 1.259 | 1.666 | Inf | 0.756 | 0.874225801 |
|  | Summer: Control vs Exclosure | -3.683 | 1.186 | Inf | -3.107 | 0.010217593 |
| SAL | Spring: Control vs Exclosure | -6.721 | 1.115 | Inf | -6.027 | 1.00E-08 |
|  | Control: Spring vs Summer | -5.537 | 0.867 | Inf | -6.383 | 1.04E-09 |
|  | Control Spring vs Exclosure Summer | -9.366 | 1.427 | Inf | -6.564 | 3.13E-10 |
|  | Exclosure Spring vs Control Summer | 1.184 | 1.398 | Inf | 0.847 | 0.831854091 |
|  | Exclosure: Spring vs Summer | -2.645 | 1.800 | Inf | -1.469 | 0.456112121 |
|  | Summer: Control vs Exclosure | -3.829 | 1.658 | Inf | -2.310 | 0.095746552 |
| JEN | Spring: Control vs Exclosure | -10.706 | 1.643 | Inf | -6.516 | 4.32E-10 |
|  | Control: Spring vs Summer | -2.144 | 0.435 | Inf | -4.926 | 5.00E-06 |
|  | Control Spring vs Exclosure Summer | -4.056 | 0.748 | Inf | -5.425 | 3.46E-07 |
|  | Exclosure Spring vs Control Summer | 8.562 | 1.690 | Inf | 5.065 | 2.44E-06 |
|  | Exclosure: Spring vs Summer | 6.649 | 1.796 | Inf | 3.701 | 0.001228134 |
|  | Summer: Control vs Exclosure | -1.913 | 0.847 | Inf | -2.258 | 0.108002574 |
| GUL | Spring: Control vs Exclosure | -3.917 | 0.763 | Inf | -5.137 | 1.67E-06 |
|  | Control: Spring vs Summer | -2.969 | 0.507 | Inf | -5.855 | 2.85E-08 |
|  | Control Spring vs Exclosure Summer | -6.497 | 1.052 | Inf | -6.175 | 3.96E-09 |
|  | Exclosure Spring vs Control Summer | 0.949 | 0.900 | Inf | 1.054 | 0.717368493 |
|  | Exclosure: Spring vs Summer | -2.580 | 1.288 | Inf | -2.003 | 0.186911296 |
|  | Summer: Control vs Exclosure | -3.528 | 1.155 | Inf | -3.054 | 0.012101607 |

**Table S6**. Pairwise comparisons of seeds produced by hand pollinated flowers outdoors. Comparisons were made from a mixed model with a zero-inflated negative binomial error distribution consisting of accession, season, exclosure treatment, all two-way interactions as fixed factors, and flat nested within block as random factors. *P*-values were adjusted using the Tukey method within each accession.

| **Accession** | **Contrast** | **Estimate** | **SE** | **df** | ***z*** | ***P*** |
| --- | --- | --- | --- | --- | --- | --- |
| HEC | Spring: Control vs Exclosure | -769.847 | 84.949 | Inf | -9.063 | 5.2958E-14 |
|  | Control: Spring vs Summer | -512.981 | 65.409 | Inf | -7.843 | 8.58E-14 |
|  | Control Spring vs Exclosure Summer | -666.690 | 80.655 | Inf | -8.266 | 4.56E-14 |
|  | Exclosure Spring vs Control Summer | 256.866 | 110.778 | Inf | 2.319 | 0.09375824 |
|  | Exclosure: Spring vs Summer | 103.157 | 113.655 | Inf | 0.908 | 0.80078479 |
|  | Summer: Control vs Exclosure | -153.709 | 100.185 | Inf | -1.534 | 0.41689778 |
| OPB | Spring: Control vs Exclosure | -772.113 | 83.518 | Inf | -9.245 | 4.341E-14 |
|  | Control: Spring vs Summer | -390.766 | 48.469 | Inf | -8.062 | 5.57E-14 |
|  | Control Spring vs Exclosure Summer | -561.743 | 76.304 | Inf | -7.362 | 1.12E-12 |
|  | Exclosure Spring vs Control Summer | 381.347 | 99.503 | Inf | 3.833 | 0.00073196 |
|  | Exclosure: Spring vs Summer | 210.370 | 110.430 | Inf | 1.905 | 0.2258433 |
|  | Summer: Control vs Exclosure | -170.978 | 87.284 | Inf | -1.959 | 0.2037524 |
| SWB | Spring: Control vs Exclosure | -578.779 | 72.442 | Inf | -7.990 | 6.195E-14 |
|  | Control: Spring vs Summer | -21.928 | 7.017 | Inf | -3.125 | 9.62E-03 |
|  | Control Spring vs Exclosure Summer | -571.166 | 72.885 | Inf | -7.837 | 8.7375E-14 |
|  | Exclosure Spring vs Control Summer | 556.851 | 72.921 | Inf | 7.636 | 1.59E-13 |
|  | Exclosure: Spring vs Summer | 7.613 | 102.070 | Inf | 0.075 | 0.99985129 |
|  | Summer: Control vs Exclosure | -549.238 | 72.979 | Inf | -7.526 | 3.40E-13 |
| BMR | Spring: Control vs Exclosure | -694.332 | 83.273 | Inf | -8.338 | 4.2411E-14 |
|  | Control: Spring vs Summer | -427.902 | 57.812 | Inf | -7.402 | 8.3356E-13 |
|  | Control Spring vs Exclosure Summer | -953.790 | 95.921 | Inf | -9.943 | 3.3973E-14 |
|  | Exclosure Spring vs Control Summer | 266.430 | 104.732 | Inf | 2.544 | 0.05342768 |
|  | Exclosure: Spring vs Summer | -259.458 | 124.280 | Inf | -2.088 | 0.15706887 |
|  | Summer: Control vs Exclosure | -525.887 | 109.323 | Inf | -4.810 | 8.97E-06 |
| LMC | Spring: Control vs Exclosure | -202.976 | 37.722 | Inf | -5.381 | 4.4344E-07 |
|  | Control: Spring vs Summer | -10.249 | 4.615 | Inf | -2.221 | 1.18E-01 |
|  | Control Spring vs Exclosure Summer | -356.911 | 54.539 | Inf | -6.544 | 3.5903E-10 |
|  | Exclosure Spring vs Control Summer | 192.727 | 38.011 | Inf | 5.070 | 2.37E-06 |
|  | Exclosure: Spring vs Summer | -153.935 | 65.804 | Inf | -2.339 | 0.08928055 |
|  | Summer: Control vs Exclosure | -346.662 | 54.640 | Inf | -6.344 | 1.34E-09 |
| PPW | Spring: Control vs Exclosure | -229.247 | 42.063 | Inf | -5.450 | 3.01E-07 |
|  | Control: Spring vs Summer | -7.934 | 5.628 | Inf | -1.410 | 4.93E-01 |
|  | Control Spring vs Exclosure Summer | -393.488 | 65.780 | Inf | -5.982 | 1.32E-08 |
|  | Exclosure Spring vs Control Summer | 221.312 | 42.449 | Inf | 5.214 | 1.11E-06 |
|  | Exclosure: Spring vs Summer | -164.242 | 77.635 | Inf | -2.116 | 0.14808177 |
|  | Summer: Control vs Exclosure | -385.554 | 65.960 | Inf | -5.845 | 3.03E-08 |
| CAV | Spring: Control vs Exclosure | -340.107 | 54.670 | Inf | -6.221 | 2.96E-09 |
|  | Control: Spring vs Summer | -18.136 | 9.115 | Inf | -1.990 | 1.92E-01 |
|  | Control Spring vs Exclosure Summer | -431.523 | 63.040 | Inf | -6.845 | 4.58E-11 |
|  | Exclosure Spring vs Control Summer | 321.971 | 55.575 | Inf | 5.793 | 4.13E-08 |
|  | Exclosure: Spring vs Summer | -91.417 | 82.876 | Inf | -1.103 | 0.68764994 |
|  | Summer: Control vs Exclosure | -413.387 | 63.489 | Inf | -6.511 | 4.47E-10 |
| MOR | Spring: Control vs Exclosure | -191.599 | 50.124 | Inf | -3.823 | 0.00076186 |
|  | Control: Spring vs Summer | -7.974 | 4.014 | Inf | -1.987 | 1.93E-01 |
|  | Control Spring vs Exclosure Summer | -304.501 | 49.231 | Inf | -6.185 | 3.72E-09 |
|  | Exclosure Spring vs Control Summer | 183.625 | 50.365 | Inf | 3.646 | 1.52E-03 |
|  | Exclosure: Spring vs Summer | -112.901 | 70.462 | Inf | -1.602 | 0.3772626 |
|  | Summer: Control vs Exclosure | -296.527 | 49.309 | Inf | -6.014 | 1.09E-08 |
| AJEN | Spring: Control vs Exclosure | -555.298 | 73.290 | Inf | -7.577 | 2.3803E-13 |
|  | Control: Spring vs Summer | -7.486 | 3.766 | Inf | -1.988 | 1.93E-01 |
|  | Control Spring vs Exclosure Summer | -622.570 | 76.153 | Inf | -8.175 | 4.9072E-14 |
|  | Exclosure Spring vs Control Summer | 547.811 | 73.417 | Inf | 7.462 | 5.39E-13 |
|  | Exclosure: Spring vs Summer | -67.272 | 105.167 | Inf | -0.640 | 0.91915904 |
|  | Summer: Control vs Exclosure | -615.083 | 76.174 | Inf | -8.075 | 5.50E-14 |
| SAL | Spring: Control vs Exclosure | -421.320 | 62.146 | Inf | -6.780 | 7.2366E-11 |
|  | Control: Spring vs Summer | -7.996 | 4.022 | Inf | -1.988 | 1.92E-01 |
|  | Control Spring vs Exclosure Summer | -644.898 | 77.710 | Inf | -8.299 | 4.4298E-14 |
|  | Exclosure Spring vs Control Summer | 413.324 | 62.298 | Inf | 6.635 | 1.95E-10 |
|  | Exclosure: Spring vs Summer | -223.577 | 98.980 | Inf | -2.259 | 0.10783097 |
|  | Summer: Control vs Exclosure | -636.902 | 77.746 | Inf | -8.192 | 4.82E-14 |
| JEN | Spring: Control vs Exclosure | -579.641 | 75.149 | Inf | -7.713 | 9.7478E-14 |
|  | Control: Spring vs Summer | -33.237 | 11.817 | Inf | -2.813 | 2.53E-02 |
|  | Control Spring vs Exclosure Summer | -935.457 | 108.450 | Inf | -8.626 | 3.4861E-14 |
|  | Exclosure Spring vs Control Summer | 546.404 | 76.253 | Inf | 7.166 | 4.67E-12 |
|  | Exclosure: Spring vs Summer | -355.817 | 131.302 | Inf | -2.710 | 0.03401799 |
|  | Summer: Control vs Exclosure | -902.221 | 108.869 | Inf | -8.287 | 4.42E-14 |
| GUL | Spring: Control vs Exclosure | -538.031 | 71.945 | Inf | -7.478 | 4.7728E-13 |
|  | Control: Spring vs Summer | -22.487 | 8.009 | Inf | -2.808 | 2.57E-02 |
|  | Control Spring vs Exclosure Summer | -696.362 | 83.628 | Inf | -8.327 | 4.2966E-14 |
|  | Exclosure Spring vs Control Summer | 515.544 | 72.469 | Inf | 7.114 | 6.79E-12 |
|  | Exclosure: Spring vs Summer | -158.332 | 109.794 | Inf | -1.442 | 0.47298147 |
|  | Summer: Control vs Exclosure | -673.875 | 83.855 | Inf | -8.036 | 5.76E-14 |
